# Discovery of Three 22-Membered Macrolides by Deciphering the Streamlined Genome of Mangrove-Derived *Streptomyces* sp. HM190

**DOI:** 10.3389/fmicb.2020.01464

**Published:** 2020-06-26

**Authors:** Yanghui Ye, Nusratgul Anwar, Xuming Mao, Shihua Wu, Cen Yan, Zhe Zhao, Ran Zhang, Yanfang Nie, Jianwei Zhang, Jidong Wang, Min Wu

**Affiliations:** ^1^Ocean College, Zhejiang University, Hangzhou, China; ^2^College of Life Sciences, Zhejiang University, Hangzhou, China; ^3^Cardiovascular Health Department, AstraZeneca Trading Co., Ltd., Wuxi, China; ^4^Key Laboratory of Vector Biology and Pathogen Control of Zhejiang Province, College of Life Science, Huzhou University, Huzhou, China

**Keywords:** *Streptomyces* sp. HM190, biosynthetic gene clusters, 22-membered macrolides, structure elucidation, cytotoxic activities

## Abstract

Strain HM190, a moderate halophile, was isolated from rhizosphere soil of the mangrove *Kandelia obovata* in Fugong village, China. The 16S ribosomal RNA (rRNA) gene sequence and the results of phylogenetic analysis revealed that strain HM190 belonged to the genus *Streptomyces* and had the highest sequence similarity of 99.79% to *Streptomyces heilongjiangensis* NEAU-W2^T^. The complete genome of strain HM190 comprised 7,762,826 bp in a linear chromosome with 71.97% G + C content. According to antiSMASH analysis, a total of 30 biosynthetic gene clusters (BGCs) were predicted to be involved in secondary metabolism, 12 of which were responsible for the production of polyketide- and non-ribosomal peptide-derived secondary metabolites. Gene cluster 5 was responsible for macrolide biosynthesis in a strain-specific 126,331-bp genomic island belonging to the left-arm region. Combined genomics–metabolomics analysis led to the discovery of three 22-membered macrolides (compounds **1**–**3**). Their structures were elucidated by using spectroscopic techniques including high-resolution electrospray ionization mass spectroscopy (HRESIMS) and nuclear magnetic resonance (NMR). The absolute configurations of compounds **1–3** were determined by the X-ray single crystal diffraction and NMR data analysis. All three compounds displayed moderate cytotoxic activities toward tumor cell lines HepG2, A549, and HCT116.

## Introduction

Mangrove ecosystems cover about 60–75% of the world’s tropical and subtropical coastlines and grow in saline coastal sediment habitats at transition zones with ocean, fresh water, and land (Holguin et al., 2001). The tidal action in these areas causes large changes in temperature, oxygen, and salinity levels during the day, which leads to the formation of unique microbial communities (Kathiresan and Bingham, 2001). The phylogenetic diversity of mangrove microbial communities has been well established based on 16S ribosomal RNA (rRNA) analytical approaches, including PCR cloning (Li et al., 2011), denaturing gradient gel electrophoresis (Tian et al., 2008), and pyrosequencing (Santos et al., 2011). Recently, various mangrove-derived microorganisms have attracted increasing attention in the drug discovery field, especially those of the genus *Streptomyces*, as important sources of biologically active compounds with diverse structures (Xu, 2011; Thatoi et al., 2013; Blunt et al., 2015).

The genus *Streptomyces* belongs to the family *Streptomycetaceae*, the order *Streptomycetales*, and the class *Actinobacteria*, and was originally described in Waksman and Henrici (1943). At the time of writing, 854 *Streptomyces* species with validly published names had been proposed according to List of Prokaryotic names with Standing in Nomenclature^1^. Some of these *Streptomyces* strains have been isolated from a wide range of marine habitats, including mangroves (Yan et al., 2010), marine sediments (Zhao et al., 2009), seawater (Zhu et al., 2011), sponges (Huang et al., 2016), algae (Girão et al., 2019), and corals (Alfredo et al., 2017). *Streptomyces* constitutes an important source of new natural products because its strains contain a large number of biosynthetic gene clusters (BGCs) associated with the production of multiple secondary metabolites (Hwang et al., 2014; Liu et al., 2018). Since the first complete genome of *Streptomyces coelicolor* A3(2) was sequenced and reported in Bentley et al. (2002), increasing numbers of *Streptomyces* genomes have been sequenced and deposited in public databases in recent years (Studholme, 2016), which has led to an increase in genomics–metabolomics studies of this genus (Xu et al., 2019). However, most of these genomes were draft genomes. The number of complete genomes of *Streptomyces* has been limited because of their high G + C content, which results in shorter reads and much higher error rates during genome sequencing (Ioanna et al., 2012; Zhong et al., 2013).

**FIGURE 1:**
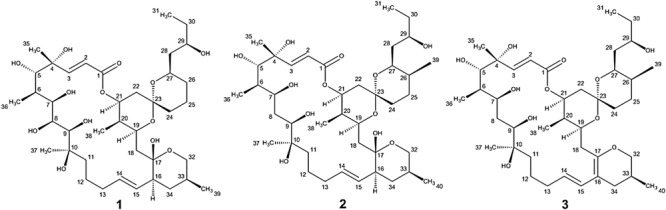
Structures of compounds **1**–**3**.

During our work focused on discovering microorganism diversity from mangrove sample, Strain HM190, as a novel species of the genus *Streptomyces*, was isolated from rhizosphere soil of the mangrove *Kandelia obovata* in Fugong village, Zhangzhou, China. The complete genome of the strain was sequenced, and 30 gene clusters were predicted to be involved in the biosynthesis of secondary metabolites using the antiSMASH 5.0 software (Blin et al., 2019). Genome analysis of the isolate indicated that gene cluster 5 was probably involved in the production of a compound structurally similar to the macrolide of apoptolidin, based on its high similarity to the type I polyketide synthase (PKS) from the apoptolidin BGC of *Nocardiopsis* sp. FU 40 (Du et al., 2011). As a result, three 22-membered macrolides (compounds **1**–**3**, Figure 1) were isolated from its fermentation broth. The absolute configuration of compound **1** was confirmed by X-ray single-crystal diffraction. The absolute configurations of compounds **2** and **3** were established through detailed nuclear magnetic resonance (NMR) data analysis in comparison with compound **1** and other related compounds. Herein, we describe the isolation, structure elucidation, and cytotoxic activity of the three compounds.

## Materials and Methods

### General Experimental Procedures

Silica gel plates HSGF254 (Yantai Chemical Industry Research Institute, Yantai, China) were used for thin-layer chromatography (TLC). Column chromatography was performed with commercial silica gel (QingDao HaiYang Chemical Group Co., QingDao, China, 200-300 mesh). High-performance liquid chromatography (HPLC) separation was performed on semipreparative HPLC (Agilent 1100, Zorbax SB-C18, 5 μm, 250 × 9.4 mm inner diameter; 1.5 mL/min; 210 nm; Agilent, Palo Alto, CA, United States). NMR spectra were measured with a Bruker DRX-400 spectrometer (Bruker, Rheinstetten, Germany). The HRESIMS spectra were taken on a Q-TOF Micro LC-MS-MS mass spectrometer (Waters Co., Milford, MA, United States). Optical rotation was measured on a Perkin-Elmer 341 polarimeter (Perkin-Elmer, Suzhou, China). IR spectra were recorded on a Nicolet Magna FT-IR 750 spectrometer (Nicolet, Tokyo, Japan). UV spectra were recorded on a Varian CARY 300 BIO spectrophotometer (Varian, Cary, NC, United States).

### Isolation and Identification of Strain HM190

Strain HM190 was isolated from a soil sample collected from rhizosphere soil of the mangrove *K. obovata* of Fugong village (117° 57′ N 24° 24′ E), in Zhangzhou, China, which was placed into clean plastic buckets and stored at 4°C until use. Serially diluted (10-fold dilutions each) samples were made and spread on the five selective isolation media by the traditional dilution-plating method: HV (humic acid-vitamin agar medium), M7 (glycerine-peptone agar medium), GS (Gauze’s modified medium no. 1), ISP2 (ISP medium no. 2), and MA (marine agar 2216 medium). All of the media were supplemented with 25 mg/L of nalidixic acid and 50 mg/L of nystatin. After 7 days of incubation at 30°C, a white-pigmented colony was picked from MA plates and named as HM190. After repeated plate streaking on the same medium, pure strains were obtained from individual colonies and preserved at −80°C as suspension with 25% (v/v) glycerol for further use (Ye et al., 2019).

For phylogenetic studies, the isolate was grown in MB (marine broth 2216 medium) for 7 days at 30°C. The 16S rRNA gene sequence amplification was performed by using universal primers 27F [5′-AGAGTTTGATCCTGGCTCAG-3′] and 1492R [5′-ACGGCTACCTTGTTACGACTT-3′] (Anzai et al., 2000). Amplification reactions were prepared in a 25 μL final reaction volume containing 12.5 μL of PCR SuperMix, 11 μL of distilled water, 0.5 μL of each primer, and 0.5 μL of extracted DNA template. PCR was performed under the following conditions: 30 cycles of 94°C/5 min, 4°C/30 s, 55°C/30 s, 72°C/75 s and a final extension of 72°C/10 min. PCR products were ligated to vector pMD 19-T (TaKaRa) and cloned into *Escherichia coli* DH5a for sequencing, and the almost-complete sequence of the 16S rRNA gene sequence was obtained. The obtained sequence was assembled with DNASTAR SeqMan (LaserGene, Madison, WI, United States). Further, the 16S rRNA gene sequence was analyzed by submitting to the NCBI^2^ and the EzTaxon-e server^3^ (Altschul et al., 1997; Yoon et al., 2017). The multiple sequences were aligned with Clustal W (Thompson et al., 1994). Phylogenetic trees were constructed with the MEGA 5.0 software package (Molecular Evolutionary Genetics Analysis, version 5.0) (Tamura et al., 2011) by using neighbor-joining (Saitou and Nei, 1987), minimum-evolution (Rzhetsky and Nei, 1992), and maximum-likelihood (Felsenstein, 1981). Bootstrap analysis (1000 replicates) was used to evaluate the trees topology. Kimura two-parameter model was used for phylogeny construction and evolutionary distances analysis (Kimura, 1981). The GenBank/EMBL/DDBJ accession number for the 16S rRNA gene sequence of strain HM190 is MN897722.

### Genome Sequencing and Annotation

For DNA isolation, strain HM190 was inoculated into MB medium and grown at 30°C with shaking (200 r/min) for 7 days. High-quality genomic DNA was extracted using the Bacteria Genomic DNA Extraction Kit (DongSheng Biotech) according to the manufacturer’s instructions. The genome was sequenced using the PacBio RS II platform and the Illumina HiSeq 4000 platform (Beijing Genomics Institute) (163-fold). Four SMRT cells Zero-Mode Wave guide arrays of sequencing were used by the PacBio platform to generate the sub-reads set. The PacBio sub-reads (length < 1 kb) were removed. To improve the accuracy of the genome sequence, the GATK^4^ and the SOAP (SOAP2, SOAPsnp, SOAPindel) tool packages were used to make single-base corrections. To confirm the presence of any plasmid, the filtered Illumina reads were mapped using the SOAP to the bacterial plasmid database^5^. The gene prediction was performed by the glimmer 3^6^ with hidden Markov models. The open reading frames (ORFs) were annotated by the Rapid Annotation using Subsystem Technology (RAST) server online (Overbeek et al., 2014). The Clusters of Orthologous Groups (COG) database was used for general function annotation. For the identification of secondary metabolism gene clusters, the antiSMASH 5.0 program was used (Blin et al., 2019). Whole Genome Shotgun project of *Streptomyces* sp. HM190 has been deposited at DDBJ/ENA/GenBank under the accession CP047318.

### Fermentation, Extraction, and Isolation of Strain HM190

Strain HM190 was inoculated into a 1000-mL flask containing 250 mL of the seed culture medium consisting of glucose (0.4%), yeast extract (0.4%), malt extract (1.0%), and CaCO_3_ (0.2%), with a pH of 7.2. After incubated at 30°C for 3 days on a rotary shaker operating at 250 r/min, the 5.0% seed culture broth was added to a 50 L fermenter (containing 30 L of fermentation medium) incubation for 7 days at 30°C. The fermentation medium component was the same as the isolation medium of MB (marine broth 2216 medium).

The final 30 L of fermentation broth was centrifuged to separate supernatant and bottomed mycelial cake. The mycelial cake was subsequently extracted with MeOH (3 L). The supernatant passed through a Diaion HP-20 resin column (Mitsubushi Chemical Co., Ltd., Tokyo, Japan) and eluted with 95% EtOH. The MeOH extract and the EtOH eluent were concentrated under reduced pressure at 55°C to yield a crude extract (30 g). The crude extract was chromatographed on a silica gel column (Qingdao Haiyang Chemical Group, Qingdao, China; 200-300 mesh) eluted with CHCl_3_/MeOH (100:0, 98:2, 95:5, 90:10, 80:20, 70:30, 60:40, and 50:50, v/v) to give six fractions (Fr.1–6) based on the TLC profiles. After the Fr.5 was concentrated under reduced pressure at 53°C and dried *in vacuo*, the material was subjected to a Sephadex LH-20 gel column (GE Healthcare, Glies, United Kingdom) and eluted with CHCl_3_/MeOH (1:1, v/v), to yield two fractions (Fr.5-1 and Fr.5-2) by using TLC detection. The Fr.5-1 was further chromatographed on a silica gel column and eluted with CHCl_3_/MeOH (100:0, 98:2, 95:5, 90:10, 85:15, 80:20, 75:25, and 70:30 v/v) to give four fractions (Fr.5-1-1–Fr.5-1-4). The Fr.5-1-1 was analyzed and purified by semi-preparative reversed-phase HPLC and eluted with CH_3_CN/H_2_O (60:40, v/v) to get compound **1** (t_R_ 28.5 min, 15.2 mg). The Fr.5-1-3 was subjected to a Sephadex LH-20 column eluted with CHCl_3_/MeOH (1:1, v/v) to give three fractions (Fr.5-1-3a, Fr.5-1-3b, and Fr.5-1-3c). The Fr.5-1-3c was further separated by semi-preparative reversed-phase HPLC and eluted with CH_3_CN/H_2_O (48:52, v/v) to get compounds **2** (t_R_ 37.0 min, 9.8 mg) and **3** (t_R_ 45.0 min, 5.8 mg), respectively.

Compound **1**: white powder; [α]${}_{\text{D}}^{25}$ = −4 (c 0.05, EtOH); IR (KBr) ν_max_ 3441.9, 2931.3, 1701.1, 1647.0, 1419.2, 1392.8, and 1287.2 cm^–1^; UV (EtOH) λ_max_ (log ε) 202 (3.93), 217 (3.82) nm; ^1^H (400 MHz) and ^13^C NMR (100 MHz) data shown in Table 1; positive HRESIMS *m/z* 765.4359 [M + Na]^+^, (calcd. for C_39_H_66_NaO_13_, 765.4396).

**TABLE 1:** The ^1^H and ^13^C NMR data for compounds **1–3** (δ in ppm).

**Position**	**1^a^ (in CDCl_3_)**		**2^a^ (in CDCl_3_)**		**3^b^ (in CD_3_OD)**	
	**δc**	**δ_H_ (*J* in Hz)**	**δc**	**δ_H_ (*J* in Hz)**	**δc**	**δ_H_ (*J* in Hz)**
1	164.9, C		165.1, C		166.9, C	
2	119.5, CH	6.16 d (15.6)	119.5, CH	6.14 d (15.6)	120.0, CH	6.04 d (15.6)
3	149.9, CH	6.85 d (15.6)	151.1, CH	6.82 d (15.6)	153.8, CH	6.96 d (15.6)
4	75.1, C		75.5, C		76.8, C	
5	79.3, CH	3.82 s	80.1, CH	3.79 br s	81.2, CH	3.75 d (11.8)
6	35.3, CH	1.62 m	39.5, CH	1.52 m	39.6, CH	1.60 m
7	77.2, CH	3.97 br s	77.7, CH	4.04 br m	77.8, CH	4.01 m
8	65.9, CH	3.90 m	35.1, CH_2_	1.27 m, 1.59 m	36.2, CH_2_	1.45 m, 1.55 m
9	73.0, CH	2.93 br s	78.4, CH	3.45 br s	78.4, CH	3.26 dd (1.9, 11.3)
10	77.1, C		75.3, C		75.7, C	
11	36.8, CH_2_	1.27 m	37.5, CH_2_	1.21 m	38.7, CH_2_	1.33m
12	21.9, CH_2_	1.45 m, 1.59 m	22.5, CH_2_	1.46 m, 1.66 m	23.8, CH_2_	1.33 m, 1.68 m
13	32.4, CH_2_	1.93 m, 2.43 br d (14.0)	32.9, CH_2_	1.96 m, 2.38 m	35.3, CH_2_	1.96 m, 2.42 m
14	132.5, CH	5.20 m	132.1, CH	5.23 m	126.2, CH	5.34 m
15	132.7, CH	5.40 m	133.0, CH	5.38 m	129.7, CH	6.35 d (15.4)
16	51.1, CH	1.98 m	50.9, CH	1.99 m	110.0, C	
17	96.9, C		97.1, C		148.8, C	
18	39.7, CH_2_	1.29 br d (13.6), 2.20 t (13.4)	39.7, CH_2_	1.33 m, 2.15 t (13.4)	32.8, CH_2_	2.18 m, 2.90 t (12.4)
19	66.1, CH	4.27 br d (12.0)	66.4, CH	4.25 m	68.9, CH	4.15 ddd (1.8, 4.3, 11.4)
20	34.3, CH	2.10 m	34.7, CH	2.00 m	34.2, CH	1.96 m
21	69.5, CH	5.30 m	69.2, CH	5.36 m	71.9, CH	5.26 m
22	35.3, CH_2_	1.62 m, 1.80 m	35.4, CH_2_	1.72 m, 1.84 m	36.8, CH_2_	1.67 m, 1.73 m
23	98.2, C		98.4, C		99.4, C	
24	34.2, CH_2_	1.46 m, 1.70 m	29.2, CH_2_	1.52 m, 1.69 m	30.7, CH_2_	1.47 m, 1.70 m
25	19.3, CH_2_	1.68 m	27.0, CH_2_	1.56 m, 1.93 m	27.8, CH_2_	1.42 m, 2.16 m
26	30.8, CH_2_	1.72 m	30.8, CH	1.62 m	32.3, CH	1.60 m
27	72.3, CH	3.97 br s	67.5, CH	3.96 br d (11.6)	69.8, CH	4.02 m
28	43.7, CH_2_	1.41 m, 1.57 m	41.6, CH_2_	1.20 m, 1.63 m	41.8, CH_2_	1.32 m, 1.63 m
29	68.3, CH	3.91 m	68.6, CH	3.86 m	71.4, CH	3.73 m
30	29.9, CH_2_	1.46 m, 1.60 m	30.3, CH_2_	1.25 m, 1.52 m	32.1, CH_2_	1.60 m
31	10.2, CH_3_	0.98 t (7.2)	10.4, CH_3_	1.00 t (7.4)	10.5, CH_3_	0.99 t (6.8)
32	66.2, CH_2_	3.57 br d (8.0)	66.3, CH_2_	3.57 m	72.4, CH_2_	3.44 t (9.8), 3.99 m
33	30.5, CH	1.30 m	30.6, CH	1.48 m	28.6, CH	1.94 m
34	33.7, CH_2_	1.41 m	34.3, CH_2_	1.41 m	30.8, CH_2_	1.29 m, 2.33 dd (5.6, 16.3)
35	28.1, CH_3_	1.38 s	27.4, CH_3_	1.40 s	27.4, CH_3_	1.34 s
36	5.6, CH_3_	0.92 d (6.8)	5.2, CH_3_	0.94 t (6.6)	6.0, CH_3_	0.95 t (6.4)
37	22.4, CH_3_	1.22 s	19.9, CH_3_	1.11 s	20.9, CH_3_	1.04 s
38	6.4, CH_3_	0.76 d (6.8)	6.3, CH_3_	0.72 d (7.2)	5.6, CH_3_	0.8 d (6.8)
39	16.9, CH_3_	0.79 d (6.4)	11.4, CH_3_	0.94 d (6.6)	11.7, CH_3_	0.95 t (6.4)
40			17.1, CH_3_	0.79 d (6.8)	17.6, CH_3_	0.99 t (6.8)

*^*a*^^1^H (400 MHz), ^13^C (100 MHz); ^*b*^^1^H (600 MHz), ^13^C (150 MHz).*

Compound **2**: white powder; [α]${}_{\text{D}}^{25}$ = −6 (c 0.05, EtOH); IR (KBr) ν_max_ 3440, 2900, 1700, 1640, 1450, 1380, 1260, 1180, 1080, and 980 cm^–1^; UV (MeOH) λ_max_ 223 nm (ε 8,900); ^1^H (400 MHz) and ^13^C NMR (100 MHz) data shown in Table 1; positive HRESIMS *m/z* 763.4546 [M + Na]^+^, (calcd. for C_39_H_66_NaO_13_, 763.4603).

Compound **3**: white powder; [α] = −5 (c 0.05, EtOH); IR (KBr) ν_max_ 3450, 2950, 1720, 1660, 1460, 1390, 1280, 1180, 1100, 980 cm^–1^; UV (MeOH) λ_max_ 247 nm (ε 13,500); ^1^H (600 MHz) and ^13^C NMR (150 MHz) data shown in Table 1; positive HRESIMS *m/z* 745.4437 [M + Na]^+^, (calcd. for C_39_H_66_NaO_13_, 745.4497). The IR and UV data of compounds **2** and **3** from Akira H. et al.

### X-Ray Crystallography

Small seed crystals of compound **1** were produced by controlling evaporation of solutions at room temperature over 2 weeks in CHCl_3_/MeOH (60:40, v/v). Re-crystallization at 4°C to induce the production of high-quality crystals for X-ray diffraction was conducted for 20 days. A suitable crystal was selected and mounted on a Bruker D8 Venture diffractometer. The crystal was kept at 170.02 K during data collection. The structure was solved with the ShelXT (Sheldrick, 2015) structure solution program using Intrinsic Phasing. Calculations were made with the ShelXL (Kratzert et al., 2015) refinement package using least squares minimization as implementation in Olex2 (Dolomanov et al., 2009). Crystallographic data (excluding structure factor) for compound **1** has been deposited with the Cambridge Crystallographic Data Center under the deposition number CCDC 1990958.

### Biological Assay

The antimicrobial activities of compounds **1**–**3** against pathogenic bacteria *Klebsiella pneumoniae*, methicillin-resistant *Staphylococcus aureus*, and pathogenic fungus *Candida albicans* were detected with the minimum inhibitory concentrations (MICs) method recommended by the Clinical and Laboratory Standards Institute (Wang et al., 2019). Amphotericin B (an antifungal antibiotic) and gentamicin (an antibacterial antibiotic) were used as a positive control.

The cytotoxic activities of compounds **1**–**3** were investigated against the human colon tumor cell line HCT116, the human lung carcinoma cell line A549, and the hepatocellular carcinoma cell line HepG2 *in vitro* by the CCK8 (cell counting kit-8) colorimetric method. The cell lines were incubated in a 5% CO_2_ incubator at 37°C for 4 h under Dulbecco’s Modified Eagle’s Medium (DMEM) solution containing 10% calf serum. The adherent cells of the logarithmic growth stage were digested and seeded in a 96-well culture plate at a density of 1 × 10^4^ cells per/well. Then the test samples and controls [Frame1] were added to the medium and cultivated for 48 h. Further the CCK8 (Dojindo, Kumamoto, Japan) reagent was added to the medium and cultured for 3 h. Cell viability was detected by the absorbance at 450 nm using a SpectraMax M5 microplate reader (Molecular Devices Inc., Sunnyvale, CA, United States) (Wang et al., 2009). Doxorubicin was used as a positive control. Cell solution (dimethylsulfoxide) was tested as a negative control. The inhibitory rate of cell proliferation was expressed as IC_50_ value.

## Results and Discussion

### 16S rRNA Gene Sequence and Phylogenetic Analysis

**FIGURE 2:**
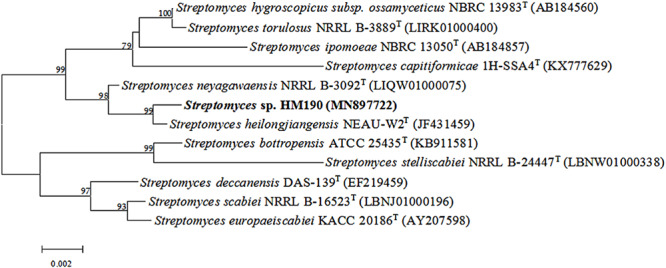
Neighbor-joining phylogenetic tree based on 16S rRNA gene sequences showing the relationships among strain HM190 and other closely related species of the genus *Streptomyces*. Bootstrap values are expressed as a percentage of 1000 replicates and only those higher than 50% are given at the branch points. Bar, 0.002 substitutions per nucleotide position.

PCR was used to determine the 16S rRNA gene sequence (1451 bp, NCBI GenBank accession number: MN897722) of strain HM190. The results of the analysis indicated that the isolate belonged to the genus *Streptomyces* and showed the highest sequence similarities to *Streptomyces heilongjiangensis* NEAU-W2^T^ (99.79%) and *Streptomyces neyagawaensis* NRRL B-3092^T^ (99.59%). In the phylogenetic analysis based on a neighbor-joining tree (Figure 2), strain HM190 fell within the cluster of the genus *Streptomyces* and formed a coherent clade with *S. heilongjiangensis* NEAU-W2^T^. The clade had firm bootstrap support and represented an independent lineage. Similar results were obtained by using the maximum-parsimony and maximum-likelihood trees (Supplementary Figures S26, S27).

### Genome Sequence and AntiSMASH Analysis

**FIGURE 3:**
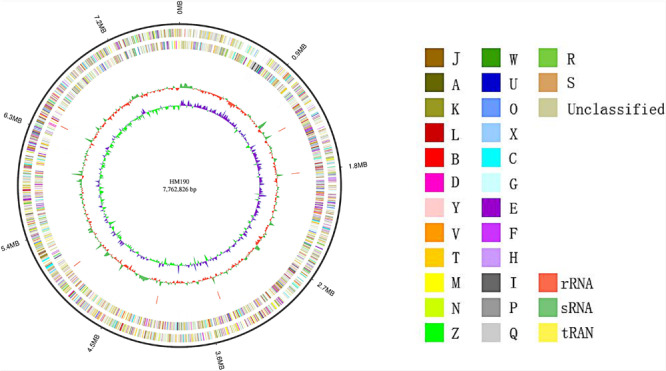
Schematic representation of the complete genome of *Streptomyces* sp. HM190. The outer scale is numbered in intervals of 0.9 Mb from the right to the left ends. From outer to inner: circle 1 (solid line) shows the genome size; in circles 2 and 3 (forward and reverse strands), the predicted protein-coding regions are colored according to clusters of orthologous groups classification (Supplementary Table S1); circles 4 and 5 (forward and reverse strands) show the distribution of rRNA operons (red), sRNA operons (green), and tRNA operons (yellow); circle 6 shows G + C content; and circle 7 indicates the GC skew.

The whole genome sequence of *Streptomyces* sp. HM190 was assembled using the PacBio RSII and Illumina HiSeq400 platforms. High-quality clean data of size 1266 Mb with a total genome size of 9.10 Mb were generated and assembled into a linear chromosome. The complete genome consisted of 776,2826 bp composing a linear chromosome with 71.97% G + C content. No plasmid was identified. A total of 6971 protein-coding genes were predicted. Of these, 4728 genes (67.8%) were annotated by querying the COG database. A map of the chromosome and the COG functional categories of strain HM190 are shown in Figure 3. For further accurate secondary metabolism BGC (SMBGC) mining analysis, 30 SMBGCs of strain HM190 were proposed using antiSMASH 5.0 (Blin et al., 2019), occupying 15.4% of the chromosome (Supplementary Table S2).

Twelve of the 30 BGCs were predicted to be responsible for the production of PKS and non-ribosome peptide- (NRPS) derived secondary metabolites, including one type I PKS (cluster 5), one type II PKS (cluster 14), two type III PKS (clusters 2, 16), two NRPS (clusters 3, 21), and six hybrid BGCs (clusters 1, 4, 6, 10, 15, 23), which possessed genes encoding more than one type of biosynthetic enzyme. Cluster 5 encoded for a type I PKS that is probably responsible for the biosynthesis of a 20/21-membered macrolide of apoptolidin. A sequence similarity of 74.5% was obtained between cluster 5 and the *Nocardiopsis* sp. FU 40 apoptolidin gene cluster (NCBI accession number: JF819834) based on BLASTN analysis. According to Du et al. (2011), the apoptolidin gene cluster of *Nocardiopsis* sp. FU 40 produces 10 apoptolidin and isoapoptolidin compounds, and the biosynthetic pathway was confirmed. Cluster 14 was probably involved in the production of a hiroshidine-like compound, based on its high similarity to the type II PKS enzyme from the hiroshidine BGC from *Streptomyces hiroshimensis* (Moon et al., 2019). Gene clusters 2 and 16 encoded, respectively, for type III PKSs that resembled the alkylresorcinol and germicidin PKSs from *Streptomyces* spp. (Funabashi et al., 2008; Becerril et al., 2018). Cluster 3 encoded for an NRPS that probably synthesized scabichelin (Kodani et al., 2013). Cluster 21 was similar to the *Salinispora tropica* CNB-440 gene cluster associated with biosynthesis of the anticancer agent salinosporamide A (Fenical et al., 2009). Based on its high similarity to the recently discovered naphthyridinomycin BGC from *Streptomyces lusitanus*, cluster 1 was probably involved in the production of a naphthyridinomycin-like compound (Pu et al., 2013). Gene clusters 4 and 15 were predicted to be responsible for biosynthesis of gaudimycin- and glycosyl ester-type compounds, respectively (Kallio et al., 2008; Wang et al., 2015). Cluster 6 encoded for a type II PKS that probably synthesized granaticin (Ichinose et al., 1998). Cluster 10 encoded for a 2*H*-furan type PKS based on its high similarity to the *Streptomyces* sp. 88-682 gene cluster (Sun et al., 2009). Six gene clusters (8, 9, 11, 20, 22, 29) within the genome of strain HM190 were observed related to saccharide biosynthesis; cluster 11 also harbored genes encoding for melanin formation. Several other secondary metabolites were potentially produced by strain HM190, including two siderophore molecules encoded by clusters 12 and 27 (cluster 12 showed high similarity to desferrioxamine B biosynthetic genes). Bacteriocin or other unspecified ribosomally synthesized and post-translationally modified peptides were encoded by clusters 13 and 25 (cluster 13 showed high similarity to bottromycin A2 biosynthetic genes), cluster 7 (ectoine), cluster 17 (lanthipeptide or lasso peptide), cluster 18 (indole), cluster 19 (halogenide), cluster 28 [linear azol(in)e-containing peptide], and clusters 24, 26, and 30 (terpenes).

### Structure Elucidation

**FIGURE 4:**
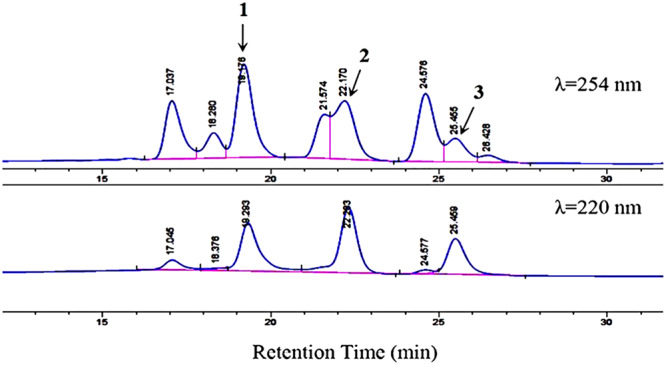
HPLC analysis of compounds **1**–**3** with a mobile phase of CH_3_CN/H_2_O (70:30, v/v). Flow rate, 1.5 mL/min. Detection wavelengths, 254 and 220 nm. The figure does not include the full chromatogram map of the crude extract but only an expansion of it.

Based on the COG categorization of the genome of strain HM190, there were a considerable number of genes related to secondary metabolite biosynthesis, transport, and catabolism (Figure 3). Together with the antiSMASH analysis, strain HM190 appeared to be an isolate with prolific potential for the production of various new and unique secondary metabolites. In order to assess this, the strain was grown for scale-up culture in 30 L of fermentation medium for 7 days at 30°C. Results of HPLC analysis of the crude extract are shown in Figure 4. Compounds **1**–**3** were purified and subjected to detailed structural analysis.

**FIGURE 5:**
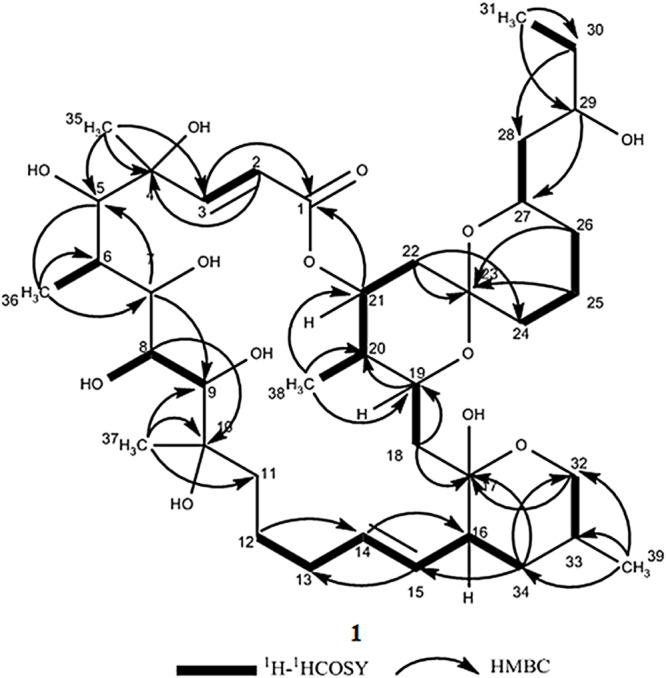
The key ^1^H-^1^H COSY and HMBC correlations for compound **1**.

Compound **1** was obtained as a white powder with a molecular formula of C_39_H_66_O_13_ (seven degrees of unsaturation) based on high-resolution electrospray ionization mass spectroscopy (HRESIMS) data (*m/z* 765.4359 [M + Na]^+^, calcd. 765.4396). The ^1^H and ^13^C NMR (Table 1), distortionless enhancement by polarization transfer (DEPT) and heteronuclear single quantum coherence (HSQC) spectra for **1** indicated the presence of two double bonds (δ_C_ 149.9, 119.5, 132.5, and 132.7); five quaternary carbons (δ_C_ 164.9, 98.2, 96.9, 77.1, and 75.1), including one carbonyl carbon (δ_C_ 164.9); six methyl groups (δ_C_ 28.1, 22.4, 16.9, 10.2, 6.4, and 5.6); 12 methylene groups, one of which was oxygenated (δ_C_ 66.2); and 16 methine groups, eight of which were oxygenated. Detailed analysis of the ^1^H–^1^H COSY (homonuclear chemical shift correlation spectroscopy) and heteronuclear multiple bond correlation (HMBC) spectra revealed that **1** was a spiroketal 22-membered macrolide with a dihydropyran moiety (Figure 5). The spiroketal ring system was established based on the HMBC correlations of H-22 with C-23, of H-22 with C-24, of H-25 with C-23, and of H-26 with C-23, together with the ^1^H-^1^H COSY correlations of H-21 with H-22, H-24 with H-25, and H-25 with H-26. The HMBC correlations from H-32 to C-17, from H-34 to C-17 and C-32, from H-34 to C-15, and from H-39 to C-32, C-33, and C-34 revealed the presence of a dihydropyran moiety and the fusion of this dihydropyran moiety to the 22-membered macrolide system. This result was supported by the ^1^H-^1^H COSY correlations H-16/H-34 and H-32/H-33. From these unambiguous one- and two-dimensional correlation NMR spectral data, the structure of **1** was assigned (Figure 1) and determined to represent a new 22-membered macrolide. These data were similar to those of phthoramycin, which was previously isolated from *Streptomyces* sp. WK-1875 (Nakagawa et al., 1989). Compound **1** had six methyl groups, whereas phthoramycin contained seven methyl groups. Comparisons of the NMR data between **1** and phthoramycin indicated that **1** differed from phthoramycin by the absence of a methyl substituent at C-26 and the presence of a hydroxy group at C-8.

The molecular formula of compound **2** was established as C_40_H_68_O_12_ (seven degrees of unsaturation) by the HRESIMS data (*m/z* 763.4546 [M + Na]^+^, calcd. 763.4603). The molecular formula of compound **2** revealed that it had the same weight as the known kaimonolide A (Akira et al., 1989). Based on detailed NMR data analysis, the structure of compound **2** was found to show the same planar structure with kaimonolide A. Compound **3** was isolated as an analog of compound **2**. The molecular formula of compound **3** was established as C_40_H_66_O_11_ (eight degrees of unsaturation) from the HRESIMS data (*m/z* 745.4437 [M + Na]^+^, calcd. 745.4497). The ^1^H and ^13^C NMR spectral data for **2** and **3** are shown in Table 1. Comparisons of the molecular formulas and NMR data of **2** and **3** indicated that **3** was a dehydrated product of **2**, resulting from the formation of a double bond at C-16/C-17. It should be noted that during our studies, compound **2** would have gradually changed to **3** in CDCl_3_ solution (Akira et al., 1989). Compounds **2** and **3** also closely resembled phthoramycin and cytovaricin B, which have been reported in *Streptomyces* spp. (Sakurai et al., 1983; Nakagawa et al., 1989).

### Absolute Configurations of Compounds **1**–**3**

**FIGURE 6:**
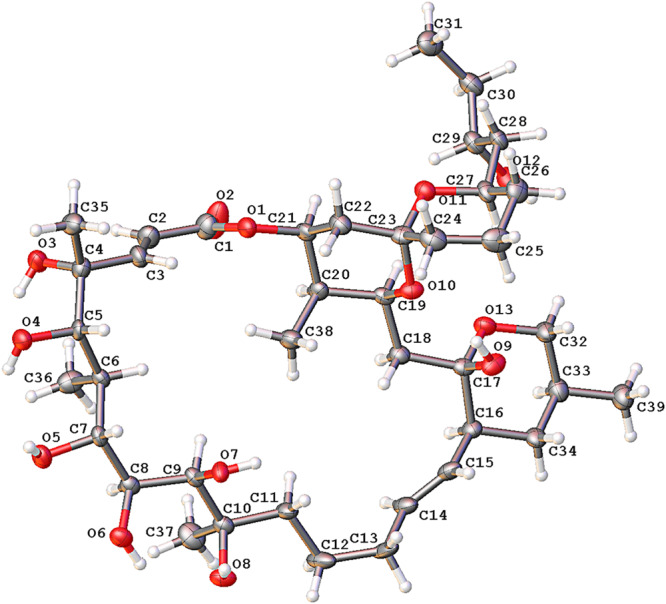
The ORTEP diagram of compound **1** obtained from X-ray data.

To define the correct absolute configuration of compound **1**, a single crystal of compound **1** was prepared for X-ray single-crystal diffraction. Crystals of compound **1** (C_39_H_66_O_13_, CCDC 1990958) were almost colorless and monoclinic with typical dimensions 0.06 × 0.06 × 0.05 mm^3^. Compound **1** crystallized in space group P21 with unit cell parameters a = 9.9665(11), b = 21.014(2), c = 21.950(2) Å, and Z = 4 molecules per unit cell. The refined structure of compound **1** had final R index [I ≥ 2sigma(I)] values of R_1_ = 0.0656, wR_2_ = 0.1426, and final R index [all data] values of R_1_ = 0.1294, wR_2_ = 0.1700. Detailed crystal data and structure refinements are shown in Table 2. The quality of the crystal was high enough for absolute configuration analysis to be carried out (Palmer and Potter, 2008). The absolute structure Flack parameter was −0.11(15), which was equal to 0.0 with standard deviation of 15 (Flack, 1983), confirming that the structure of **1** had been assigned the correct absolute configuration. Figure 6 shows the ORTEP diagram of compound **1** according to the X-ray data.

**TABLE 2:** Crystal data and structural refinement for compound **1**.

**Empirical formula**	**C_39_H_66_O_13_**
Formula weight	742.91
Temperature (K)	170.02
Crystal system	Monoclinic
Space group	P21
Unit cell dimensions	a = 9.9665(11) Å, α = 90° b = 21.014(2) Å, β = 96.321(3) c = 21.950(2) Å, γ = 90°
Volume (Å^3^)	4569.3(8)
Z	4
Calculated density (Mg/m^3^)	1.080
Absorption coefficient (mm^–1^)	0.422
F(000)	1616.0
Crystal size (mm^3^)	0.06 × 0.06 × 0.05
Wavelength (Å)	1.34139
Theta range for data collection	7.32–109.99°
Index ranges	−12 ≤ h ≤ 12, −25 ≤ k ≤ 25, -26 ≤ l ≤ 25
Reflections collected	42,745
Independent reflections	17,092 [R(int) = 0.0883, R(sigma) = 0.1157]
Data/restraints/parameters	17,092/18/964
Goodness-of-fit on F^2^	0.997
Final R indices [I ≥ 2sigma(I)]	R_1_ = 0.0656, wR_2_ = 0.1426
Final R indices [all data]	R_1_ = 0.1294, wR_2_ = 0.1700
Largest diff. peak and hole (e Å^–3^)	0.23 and −0.25
Flack parameter	−0.11(15)

Compound **2** was dehydrated at C16/C-17 to produce compound **3**. Thus, we determined the absolute configurations of compounds **2** and **3** simultaneously. Based on the similarity of the NMR spectra of compounds **1–3**, including chemical shifts and peak multiplicities (Table 1), the multiple stereocenters of **2** and **3** had the same absolute configuration as in compound **1**, although within differences at C-26 and C-27, because of the addition of a methyl substituent at C-26. According to Bilyk et al. in 2019, the absolute configurations of 22- to 26-membered macrolides compounds were confirmed by the data of NMR spectra, single-crystal X-ray crystallography, and elegant total synthesis of representative. Detailed NMR data comparison revealed a striking structural similarity of compounds **2**, **3** to kaimonolide A (Akira et al., 1989; Bilyk et al., 2019) and cytovaricin B (Sakurai et al., 1983; Yamashita et al., 1997). Finally, the absolute configurations of compounds **2** and **3** were established as shown in Figure 1.

### Biosynthetic Pathway Analysis

**FIGURE 7:**
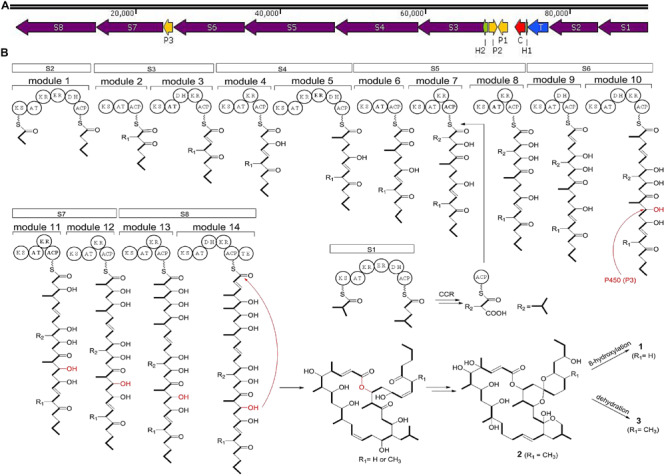
**(A)** Biosynthetic gene clusters of compounds **1**–**3** from strain HM190. S1–S8 include the PKS genes; P1–P3 account for the cytochrome P450 genes; C is a crotonyl-CoA reductase gene; T is a transcriptional regulator gene; H1 and H2 are hypothetical protein genes. **(B)** The proposed biosynthetic pathway for compounds **1–3**. ACP, acyl carrier protein; KS, ketosynthase; AT, acyltransferase; KR, ketoreductase; ER, enoylreductase; DH, dehydratase; TE, thioesterase.

In strain HM190, the predicted BGC of macrolide compounds **1**–**3** contained 15 individual ORFs, including eight type I PKS genes, three cytochrome P450 genes, one crotonyl-CoA reductase (CCR) gene, one transcriptional regulator gene, and two hypothetical protein genes (Du et al., 2011; Bilyk et al., 2019). PKS genes S1–S8 were proposed to be responsible for the biosynthesis of the polyketide core of compounds **1**–**3**. A suggested module and domain organization and a proposed model for the PKS template assembly of the macrolide backbone are shown in Figure 7. PKS gene S2 encoded the putative protein possessing the probable initiating module. The next 13 extension modules were proposed to be encoded by genes S3–S8 and were ordered according to the predicted linear arrangement of the required domains. Finally, the terminating module protein was identified to be encoded by gene S8 by its terminal punctuation with a thioesterase domain. One additional PKS gene S1 containing a complete module sequence “KS-AT-KR-ER-DH-ACP” and CCR apparently encoded a free-standing isobutylmalonyl-ACP that assembled to the macrolide backbone of module 7 in gene S5. After the core skeletons were constructed, the structural diversity of both macrocyclization and spiroketalization was mainly due to the tailoring reactions of cytochrome P450 genes to complete the hydroxylation reactions.

### Biological Activities

The cytotoxic activities of compounds **1**–**3** against HepG2, A549, and HCT116 human tumor cell lines are shown in Table 3. All the compounds suppressed the proliferation of the tested cells but exhibited different levels of activity. Compound **1** showed stronger cytotoxic activities than compounds **2** and **3**, with a half maximal inhibitory concentration value (IC_50_) of 8.76 μg/mL against hepatocellular carcinoma cell line HepG2, with IC_50_ of 15.97 μg/mL against lung carcinoma cell line A549, and with IC_50_ of 22.13 μg/mL against colon tumor cell line HCT116.

**TABLE 3:** Cytotoxic activities of compounds **1**–**3** against selected human tumor cell lines.

**Compound**	**IC_50_ (μg/mL)**		
	**HepG2**	**A549**	**HCT116**
1	8.76	15.97	22.13
2	10.52	19.63	28.25
3	12.69	20.17	29.70
Doxorubicin	0.90	0.95	1.04

The antibacterial and antifungal activities of the three compounds were detected against three human pathogens: Gram-positive bacterium methicillin-resistant *S. aureus*, Gram-negative bacterium *K. pneumoniae*, and fungus *C. albicans*. The MICs of compounds **1**–**3** were found to be >10 mg/mL, indicating that the three compounds had no biological activities against the tested pathogens.

## Conclusion

During a study of microorganism diversity in mangrove samples, a white-pigmented colony was picked and named HM190. Detailed 16S rRNA gene sequencing and phylogenetic analysis identified the strain as *Streptomyces*. The whole-genome sequence of strain HM190 was obtained using the PacBio RSII and the Illumina HiSeq 4000 platforms by long-read single-molecule real-time sequencing technology. The assembled genome comprised a linear chromosome as a single contig of 7,762,826 bp with 71.97% G + C content. Based on the results of antiSMASH analysis, a total of 30 gene clusters were predicted to be involved in the biosynthesis of secondary metabolites; 12 of the 30 BGCs were responsible for the production of PKS- and NRPS-derived secondary metabolites. Several other secondary metabolites were also predicted by their BGCs. Gene cluster 5 was predicted to be involved in the production of an apoptolidin-like macrolide based on its high similarity of 74.5% to the type I PKS enzyme from the macrolide BGC of *Nocardiopsis* sp. FU 40 (Du et al., 2011). Furthermore, a chemical investigation was carried out on strain HM190, which led to the isolation of three 22-membered macrolides (compounds **1**–**3**). The structures of the three compounds were determined based on the HRESIMS and NMR data. The absolute configurations of compounds **1**–**3** were established by the X-ray single-crystal diffraction and NMR data analysis. All three compounds showed moderate cytotoxic activities against hepatocellular carcinoma cell line HepG2, human lung carcinoma cell line A549, and human colon tumor cell line HCT116. A biosynthetic pathway for the three compounds was proposed on basis of the PKS gene cluster analysis.

## Data Availability Statement

The datasets generated for this study can be found in the GenBank/EMBL/DDBJ and accession number for the 16S rRNA gene sequence of strain HM190 is MN897722. Whole Genome Shotgun project of *Streptomyces* sp. HM190 has been deposited at DDBJ/ENA/GenBank under the accession CP047318.

## Ethics Statement

All studies of this article were processed with the standard of biosecurity and institutional safety procedures.

## Author Contributions

YY conducted and performed the experiments and prepared the manuscript. JW and MW designed the experiments. XM and SW determined the compounds structures and the biosynthetic pathway analysis. NA performed the biological activities experiments. ZZ and RZ undertook the genome analysis. CY and YN performed the microbiology experiments. JZ revised the manuscript. All authors contributed to the article and approved the submitted version.

## Conflict of Interest

JZ was employed by AstraZeneca Trading Co., Ltd.

The remaining authors declare that the research was conducted in the absence of any commercial or financial relationships that could be construed as a potential conflict of interest.

## Footnotes

1 http://www.bacterio.net/streptomyces.html

2 https://blast.ncbi.nlm.nih.gov/Blast.cgi

3 http://eztaxon-e.ezbiocloud.net

4 https://www.broadinstitute.org/gatk/

5 http://www.ebi.ac.uk/genomes/plasmid.html

6 http://ccb.jhu.edu/software/glimmer/index.shtml

## Supplementary Material

The following are available online: the HRESIMS and NMR spectra (Supplementary Figures S1–S25) of compounds **1–3**, the maximum-parsimony and maximum-likelihood phylogenetic trees (Supplementary Figures S26, S27) of strain HM190, the cluster of orthologous groups (COG) classification (Supplementary Table S1) and the antiSMASH-predicted BGCs (Supplementary Table S2) of strain HM190, and the crystal data (Supplementary Tables S3–S5) of compound **1**.
