# Supplementary material for: Discovery of Three 22-Membered Macrolides by Deciphering the Streamlined Genome of Mangrove-Derived Streptomyces sp. HM190
Source: Front Microbiol. 2020 Jun 26;11:1464. doi: 10.3389/fmicb.2020.01464 (PMC7333363; doi:10.3389/fmicb.2020.01464)
Supplement: Supplementary file 1 [file Data_Sheet_1.DOCX]

**
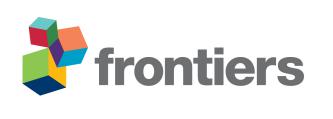
**Supplementary Material

Contents

**[Figure S1.](#_bookmark0)** [The](#_bookmark0)[HRESIMS data of compound](#_bookmark0) **[1](#_bookmark0)** [3](#_bookmark0)

**[Figure S2.](#_bookmark1)** [The IR spectrum of compound](#_bookmark1) **[1](#_bookmark1)** [4](#_bookmark1)

**[Figure S3.](#_bookmark1)** [The UV spectrum of compound](#_bookmark1) **[1](#_bookmark1)** [4](#_bookmark1)

**[Figure S4.](#_bookmark2)** [The](#_bookmark2)[1H NMR spectrum of compound](#_bookmark2) **[1](#_bookmark2)** [in CDCl](#_bookmark2)_[3](#_bookmark2)_ [(400 MHz) 5](#_bookmark2)

**[Figure S5.](#_bookmark3)** [The](#_bookmark3)[13C NMR spectrum of compound](#_bookmark3) **[1](#_bookmark3)** [in CDCl](#_bookmark3)_[3](#_bookmark3)_ [(100 MHz) 5](#_bookmark3)

**[Figure S6.](#_bookmark4)** [The DEPT135 spectrum of compound](#_bookmark4) **[1](#_bookmark4)** [in CDCl](#_bookmark4)_[3](#_bookmark4)_ [(400 MHz) 6](#_bookmark4)

**Figure S7.** The HSQC spectrum of compound **1** in CDCl_3_ (400 MHz) 6

**Figure S8-1.** The HMBC spectrum of compound **1** in CDCl_3_ (400 MHz) 7

**Figure S8-2.** The HMBC spectrum (magnification) of compound **1** in CDCl_3_ (400 MHz) 7

**Figure S8-3.** The HMBC spectrum (magnification) of compound **1** in CDCl_3_ (400 MHz) 8

**Figure S9.** The 1H-1H COSY spectrum of compound **1** in CDCl_3_ (400 MHz) 8

**Figure S10.** The NOSEY spectrum of compound **1** in CDCl_3_ (400 MHz) 9

**Figure S11.** The key NOESY correlations for compound **1** 9

**Figure S12.** The HRESIMS data of compound **2** 10

**Figure S13.** The 1H NMR spectrum of compound **2** in CDCl_3_ (400 MHz) 11

**Figure S14.** The 13C NMR spectrum of compound **2** in CDCl_3_ (100 MHz) 11

**Figure S15.** The DEPT135 spectrum of compound **2** in CDCl_3_ (400 MHz) 12

**Figure S16.** The HSQC spectrum of compound **2** in CDCl_3_ (400 MHz) 12

**Figure S17-1.** The HMBC spectrum of compound **2** in CDCl_3_ (400 MHz) 13

**Figure S17-2.** The HMBC spectrum (magnification) of compound **2** in CDCl_3_ (400 MHz) 13

**Figure S17-3.** The HMBC spectrum (magnification) of compound **2** in CDCl_3_ (400 MHz) 14

**Figure S18.** The 1H-1H COSY spectrum of compound **2** in CDCl_3_ (400 MHz) 14

**Figure S19.** The HRESIMS data of compound **3** 15

**Figure S20.** The 1H NMR spectrum of compound **3** in CD_3_OD (600 MHz) 16

**Figure S21.** The 13C NMR spectrum of compound **3** in CD_3_OD (150 MHz) 16

**Figure S22.** The DEPT135 spectrum of compound **3** in CD_3_OD (600 MHz) 17

**Figure S23.** The HSQC spectrum of compound **3** in CD_3_OD (600 MHz) 17

**Figure S24-1.** The HMBC spectrum of compound **3** in CD_3_OD (600 MHz) 18

**Figure S24-2.** The HMBC spectrum (magnification) of compound **2** in CDCl_3_ (400 MHz) 18

**Figure S24-3.** The HMBC spectrum (magnification) of compound **2** in CDCl_3_ (400 MHz) 19

**Figure S25.** The 1H-1H COSY spectrum of compound **3** in CD_3_OD (600 MHz) 19

**Figure S26.** The maximum-parsimony tree of strain HM190 20

**Figure S27.** The maximum-likelihood tree of strain HM190 20

**Table S1.** The clusters of orthologous groups (COG) classification of the complete genome of

strain HM190.. 21

**Table S2.** The antiSMASH-predicted BGCs for [strain](https://www.ezbiocloud.net/taxonomy?tn=Streptomyces heilongjiangensis)HM190. 22

**Table S3.** Fractional atomic coordinates (×10^4^) and equivalent isotropic displacement parameters

(Å^2^×10^3^) for compound **1** by the X-ray crystallography......................................................................27

**Table S4.** Bond lengths (Å) for compound **1** by the X-ray crystallography........................................30

**Table S5.** Bond angles (˚) for compound **1** by the X-ray crystallography.. .33


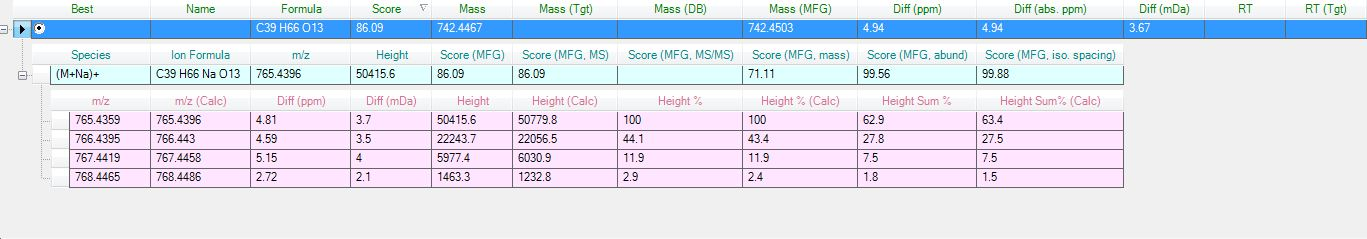


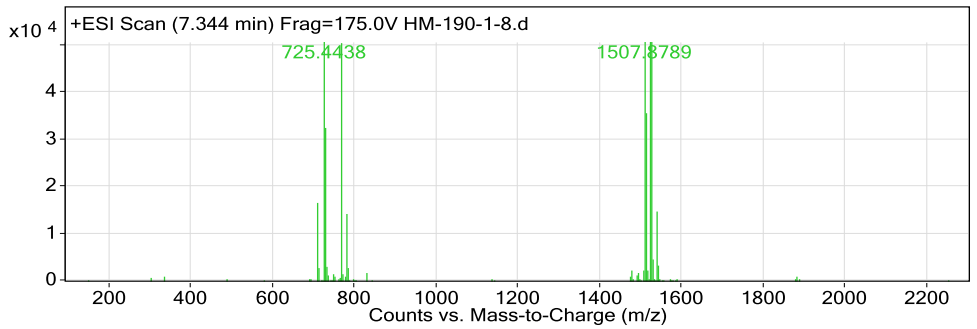

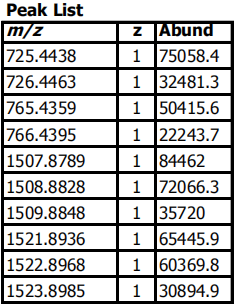


**Figure S1.** The HRESIMS data of compound **1**


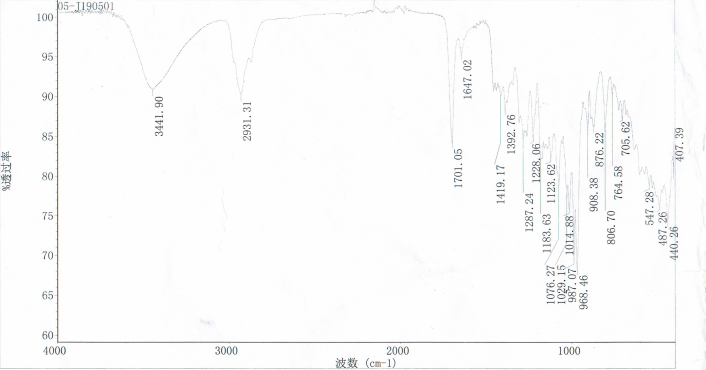


**Figure S2.** The IR spectrum of compound **1**


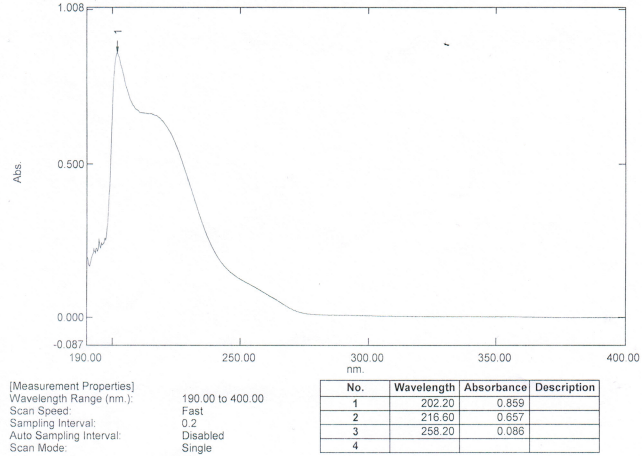


**Figure S3.** The UV spectrum of compound **1**


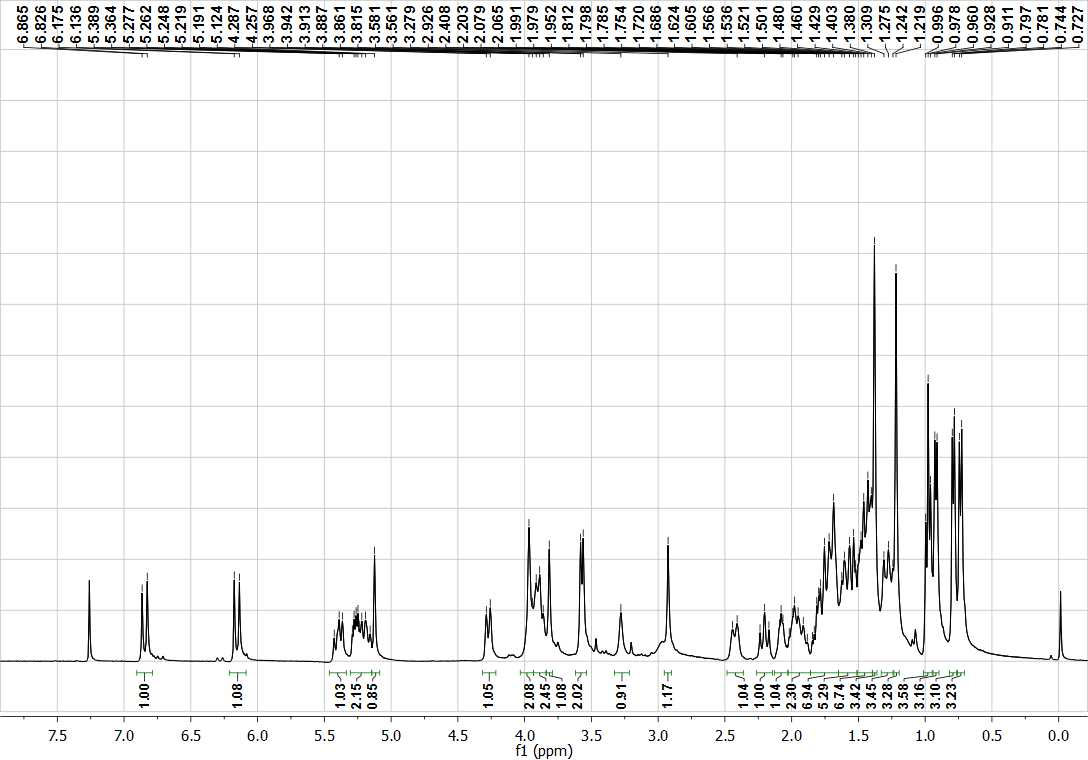


**Figure S4.** The 1H NMR spectrum of compound **1** in CDCl_3_ (400 MHz)


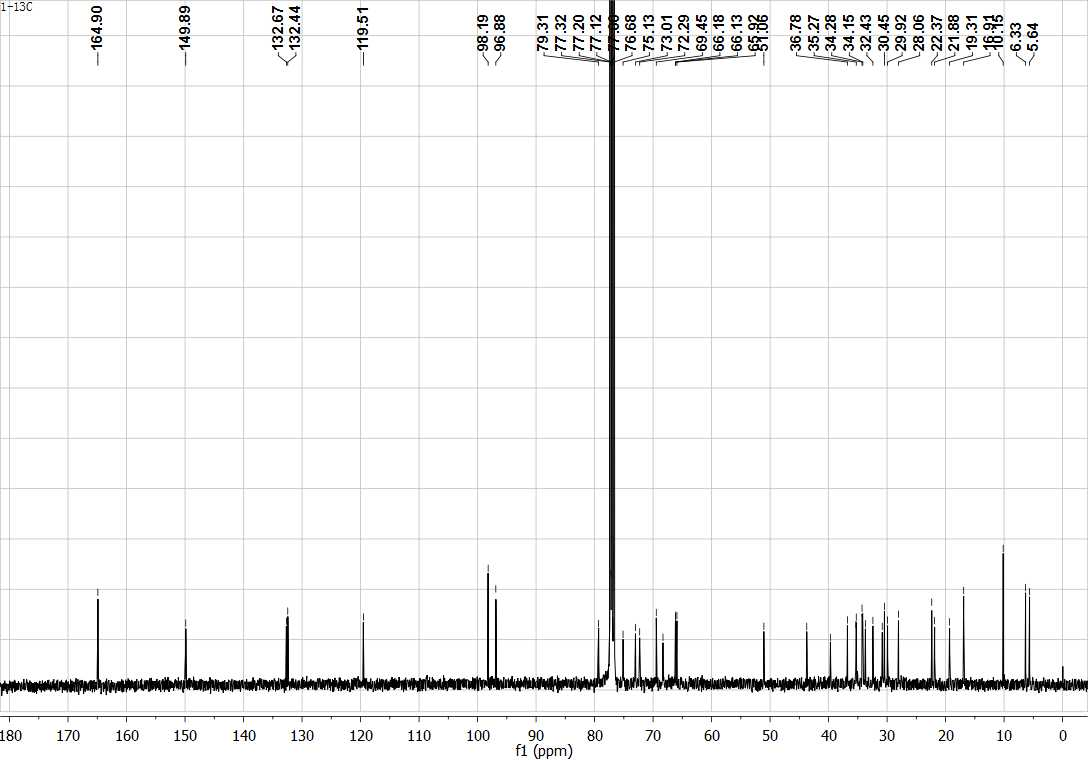


**Figure S5.** The 13C NMR spectrum of compound **1** in CDCl_3_ (400 MHz)


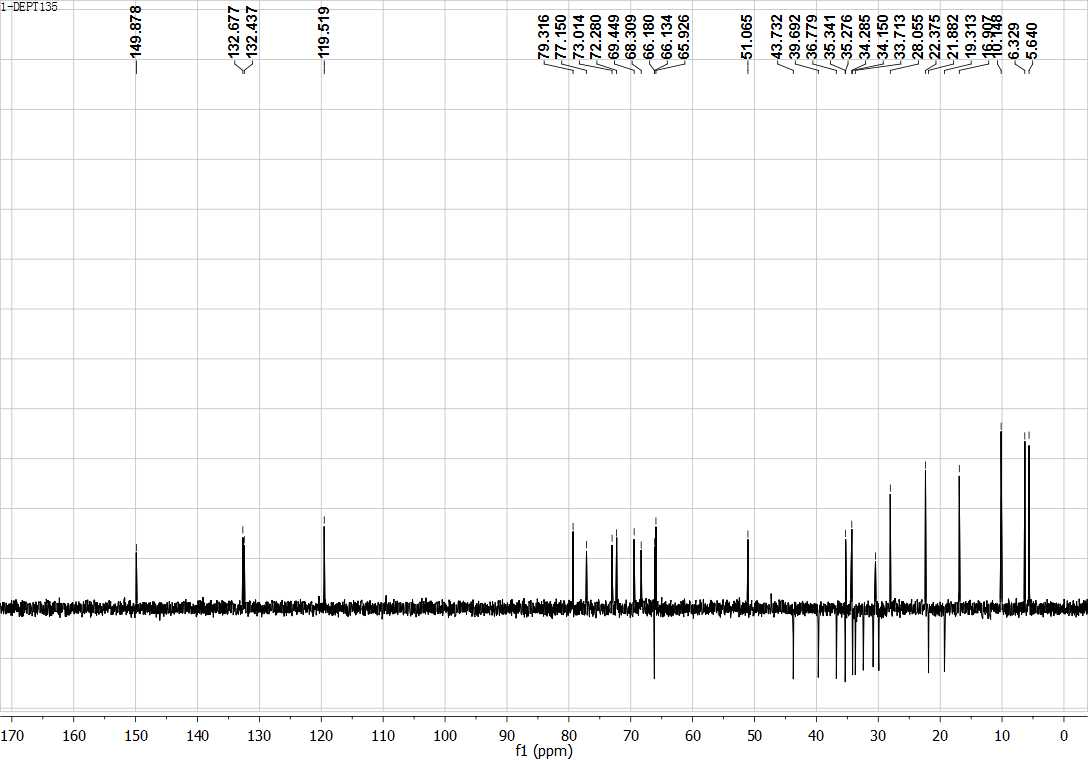


**Figure S6.** The DEPT135 spectrum of compound **1** in CDCl_3_ (400 MHz)


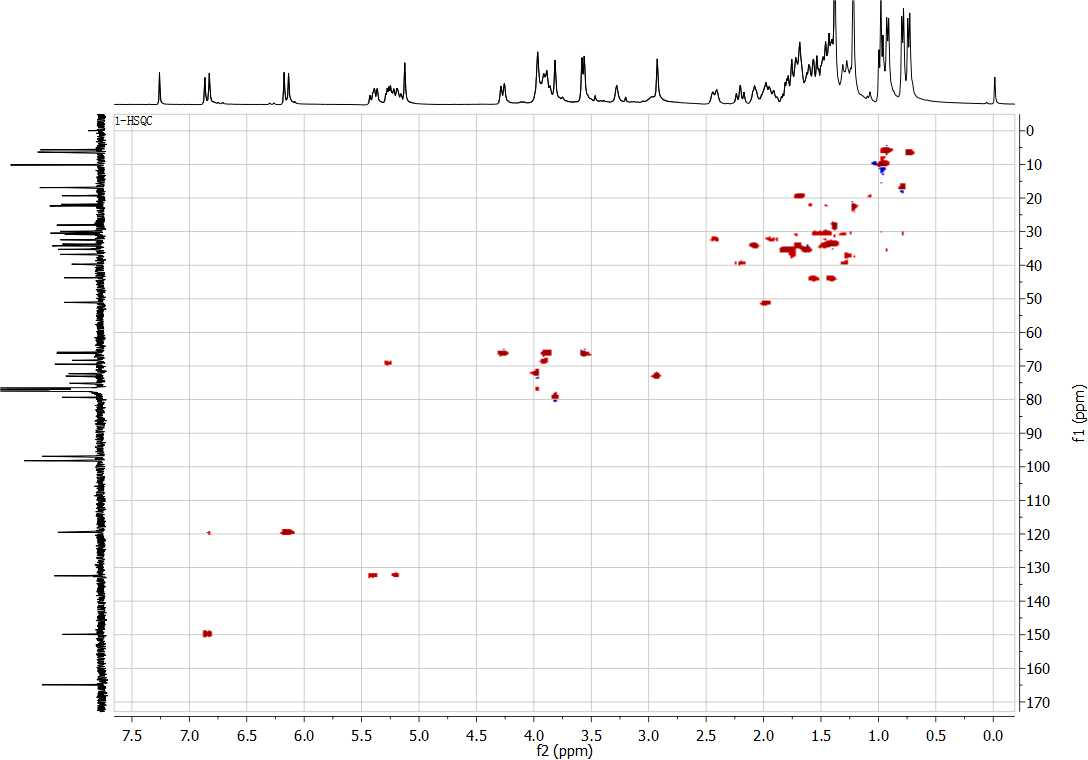


**Figure S7.** The HSQC spectrum of compound **1** in CDCl_3_ (400 MHz)


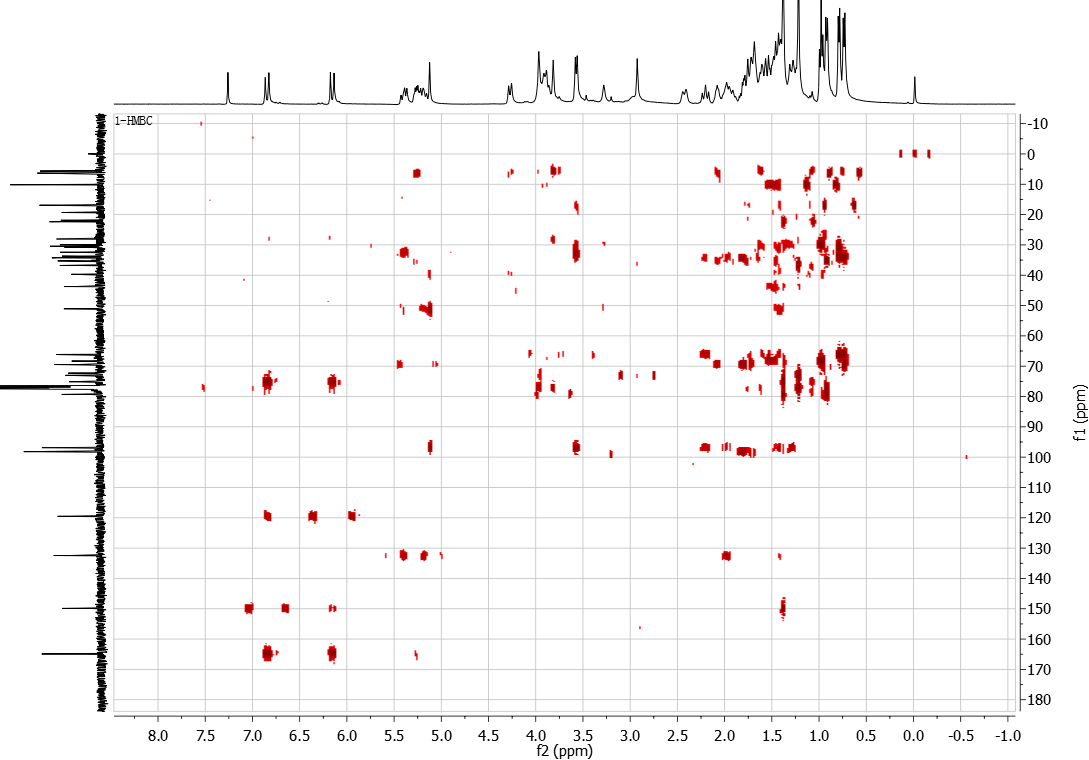


**Figure S8-1.** The HMBC spectrum of compound **1** in CDCl_3_ (400 MHz)


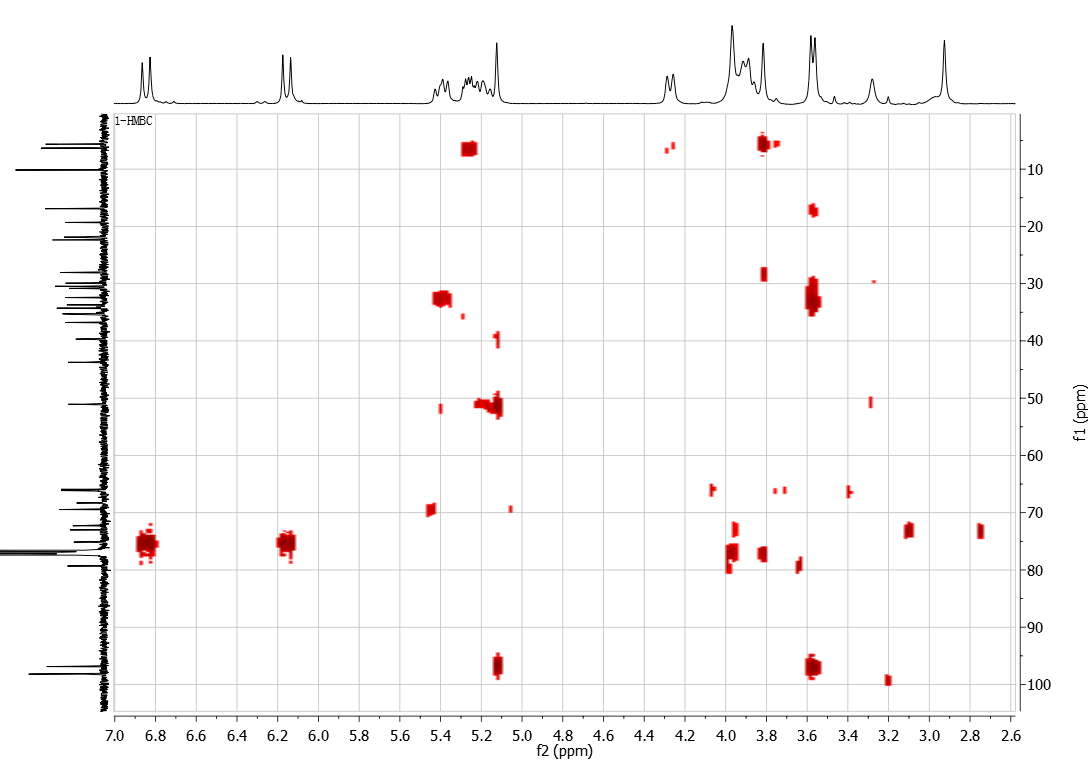


**Figure S8-2.** The HMBC spectrum of compound **1** in CDCl_3_ (400 MHz)


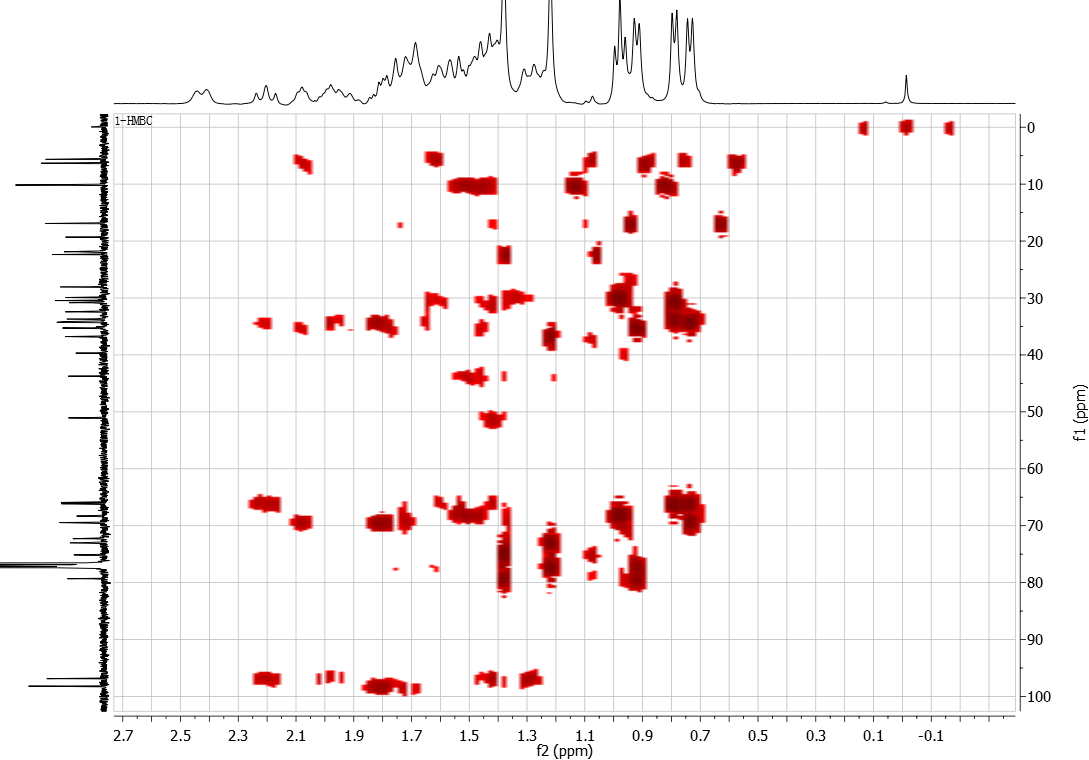


**Figure S8-3.** The HMBC spectrum of compound **1** in CDCl_3_ (400 MHz)


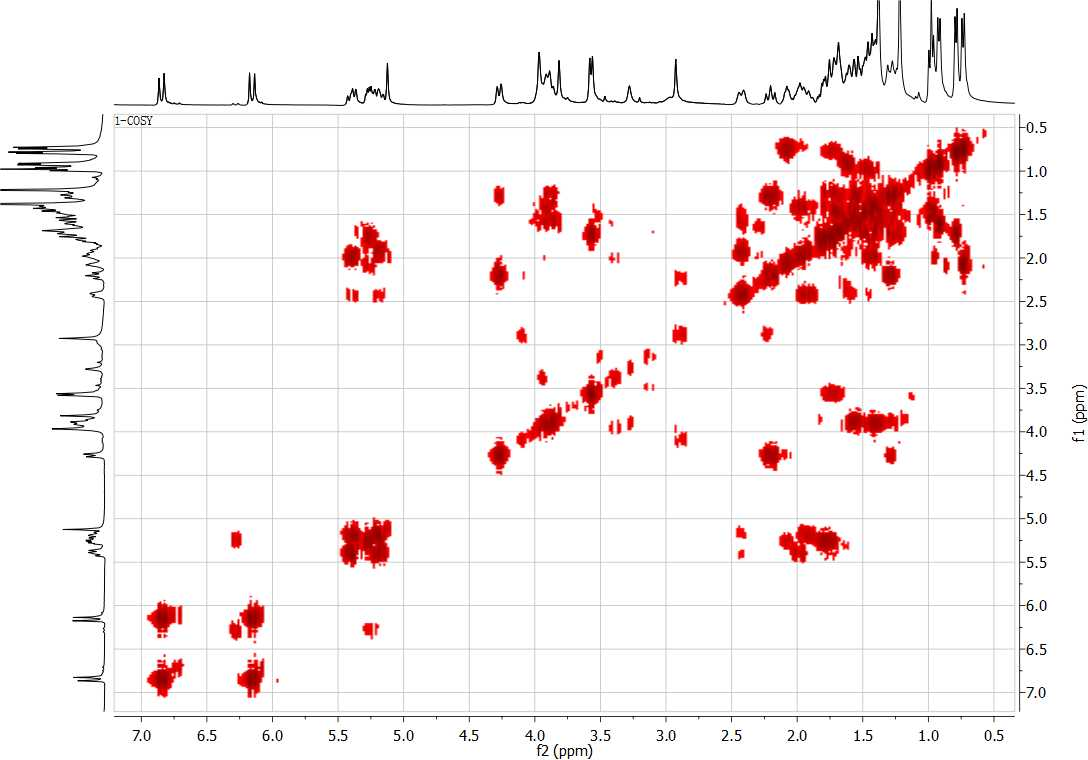


**Figure S9.** The ^1^H-^1^H COSY spectrum of compound 1 in CDCl_3_ (400 MHz)

**
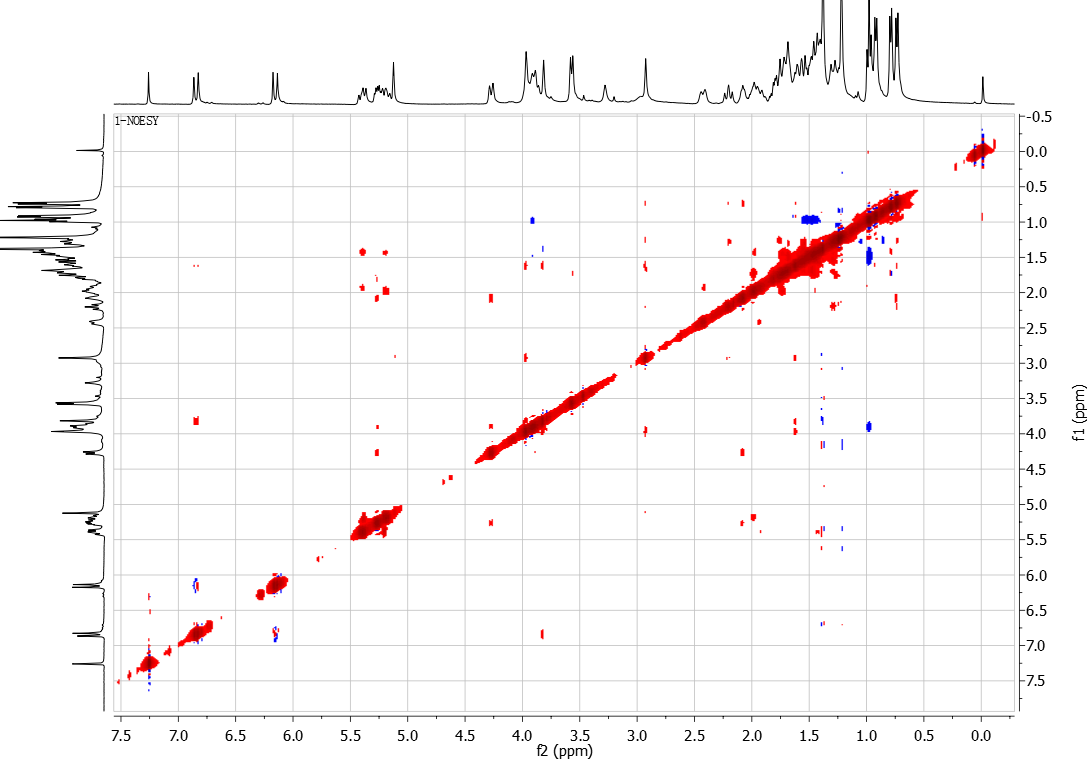
**

**Figure S10.** The NOSEY spectrum of compound **1** in CDCl_3_ (400 MHz)

**Figure S11.** The key NOESY correlations for compound **1**


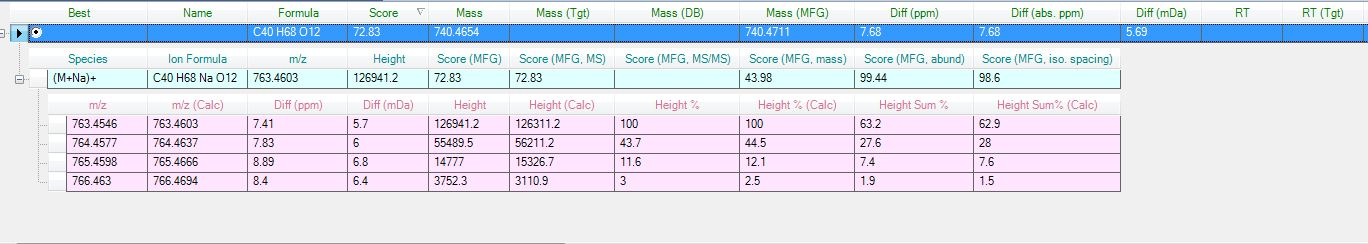


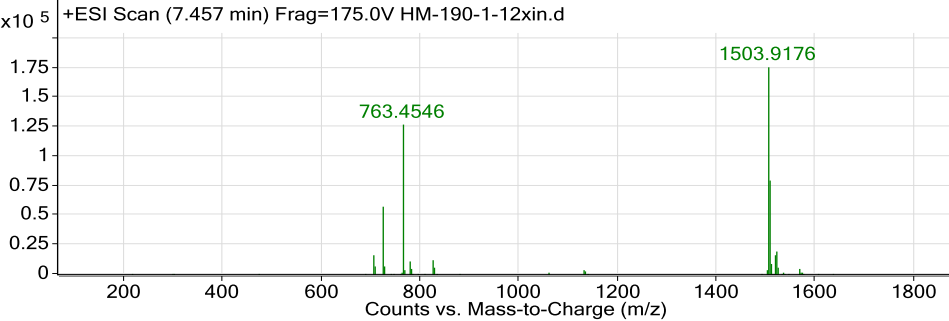

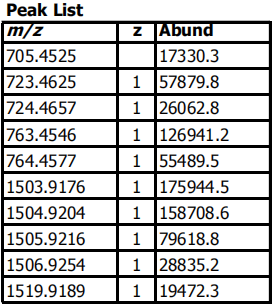


**Figure S12.** The HRESIMS data of compound **2**


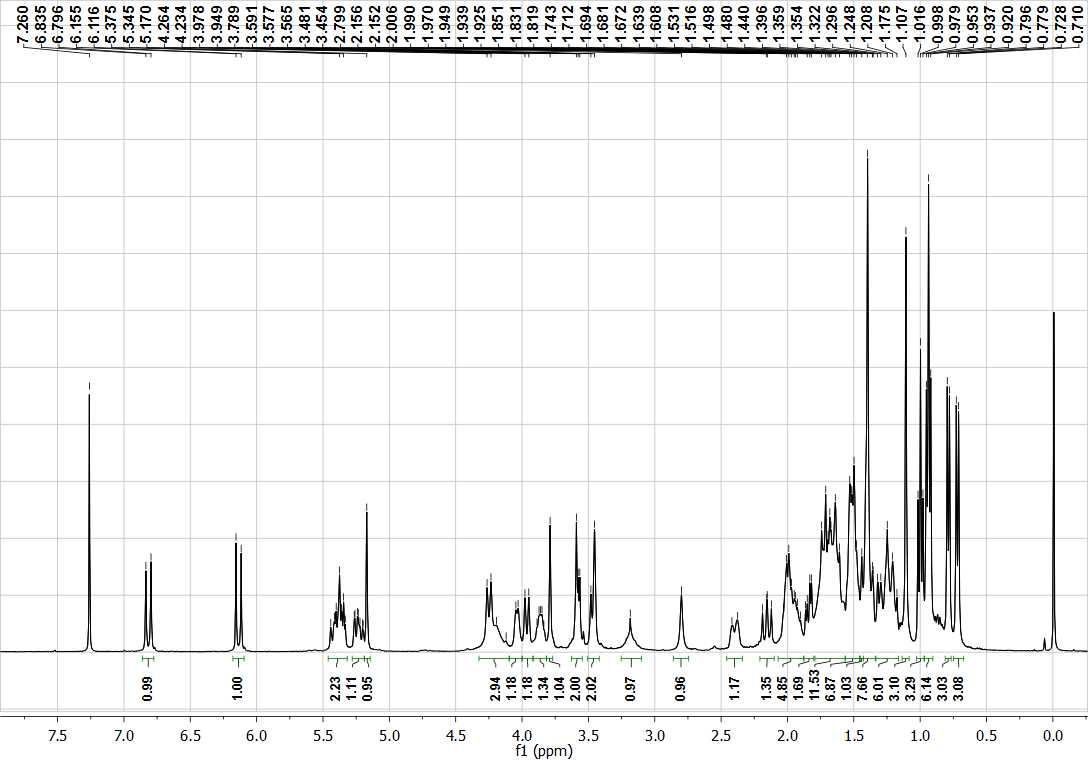


**Figure S13.** The 1H NMR spectrum of compound **2** in CDCl_3_ (400 MHz)


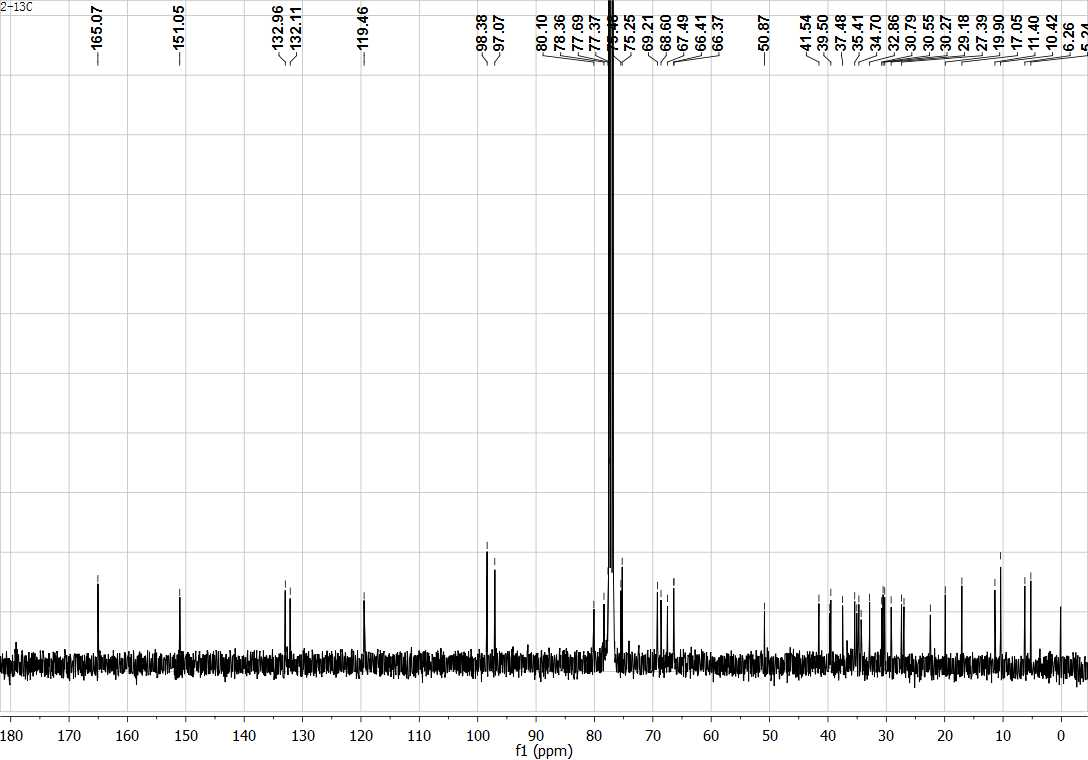


**Figure S14.** The 13C NMR spectrum of compound **2** in CDCl_3_ (400 MHz)


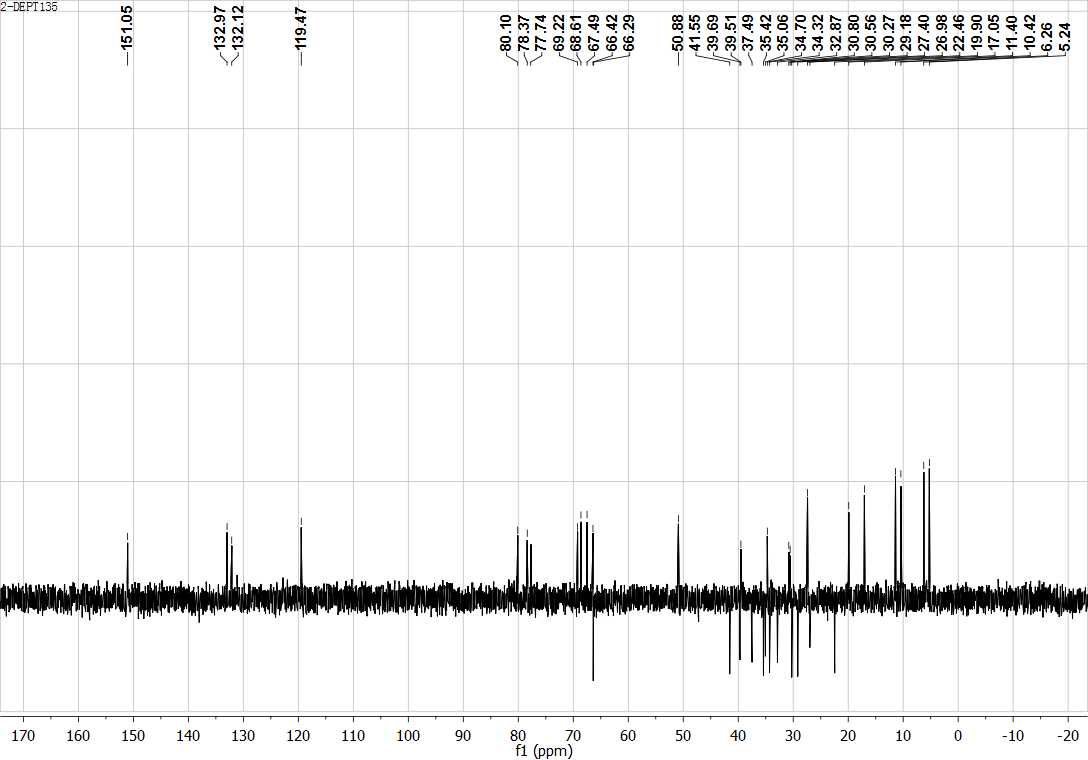


**Figure S15.** The DEPT135 spectrum of compound **2** in CDCl_3_ (400 MHz)


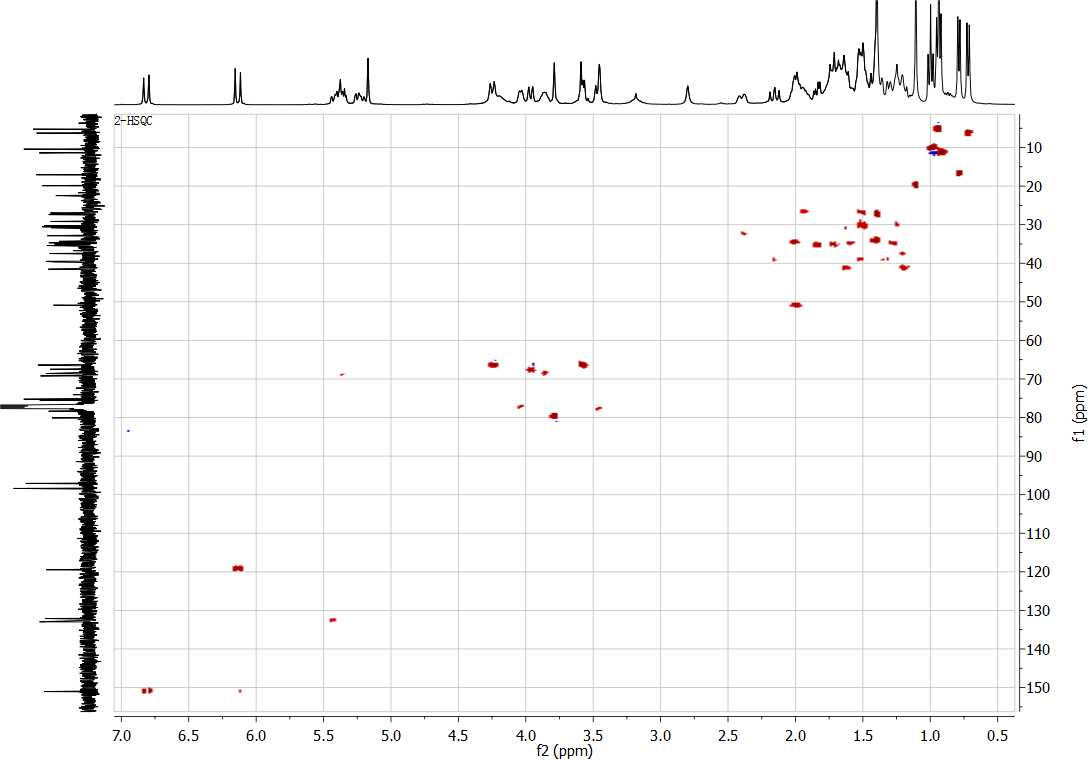


**Figure S16.** The HSQC spectrum of compound **2** in CDCl_3_ (400 MHz)


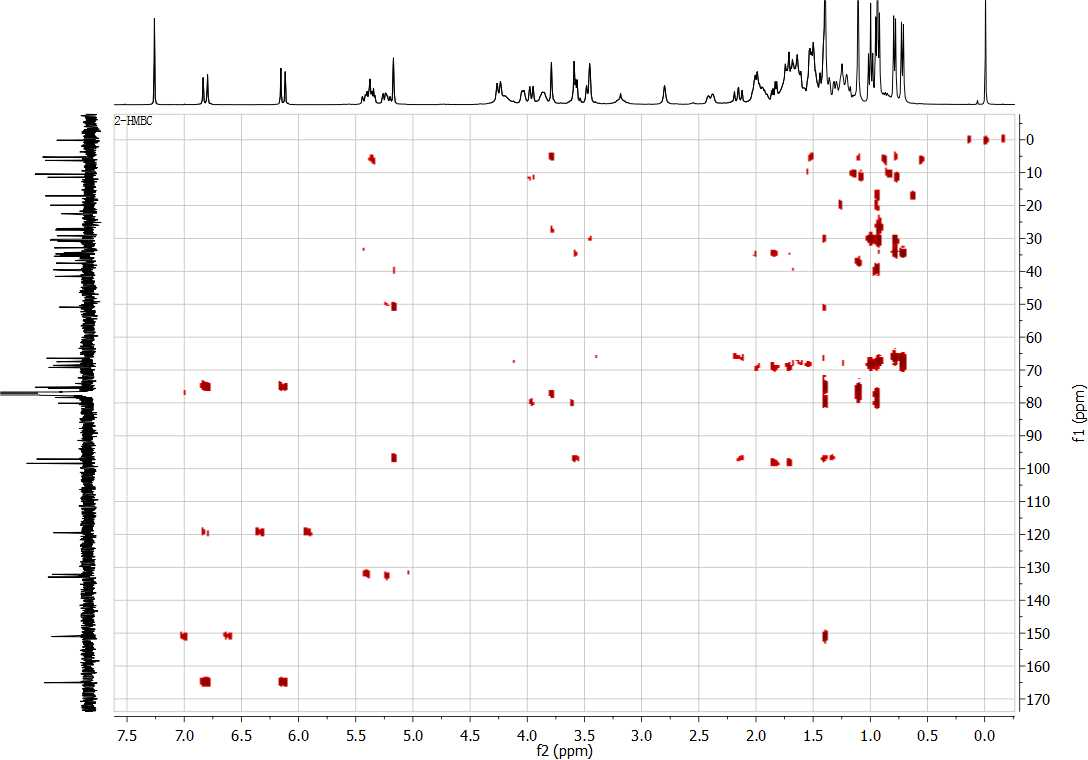


**Figure S17-1.** The HMBC spectrum of compound **2** in CDCl_3_ (400 MHz)


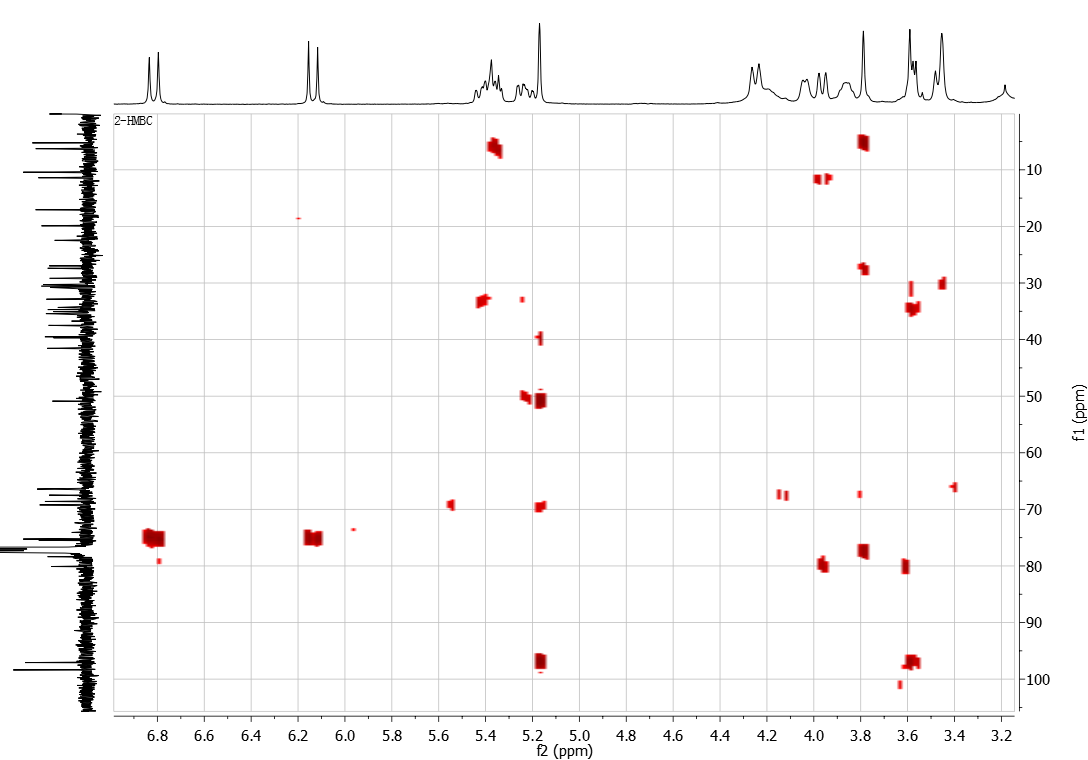


**Figure S17-2.** The HMBC spectrum of compound **2** in CDCl_3_ (400 MHz)


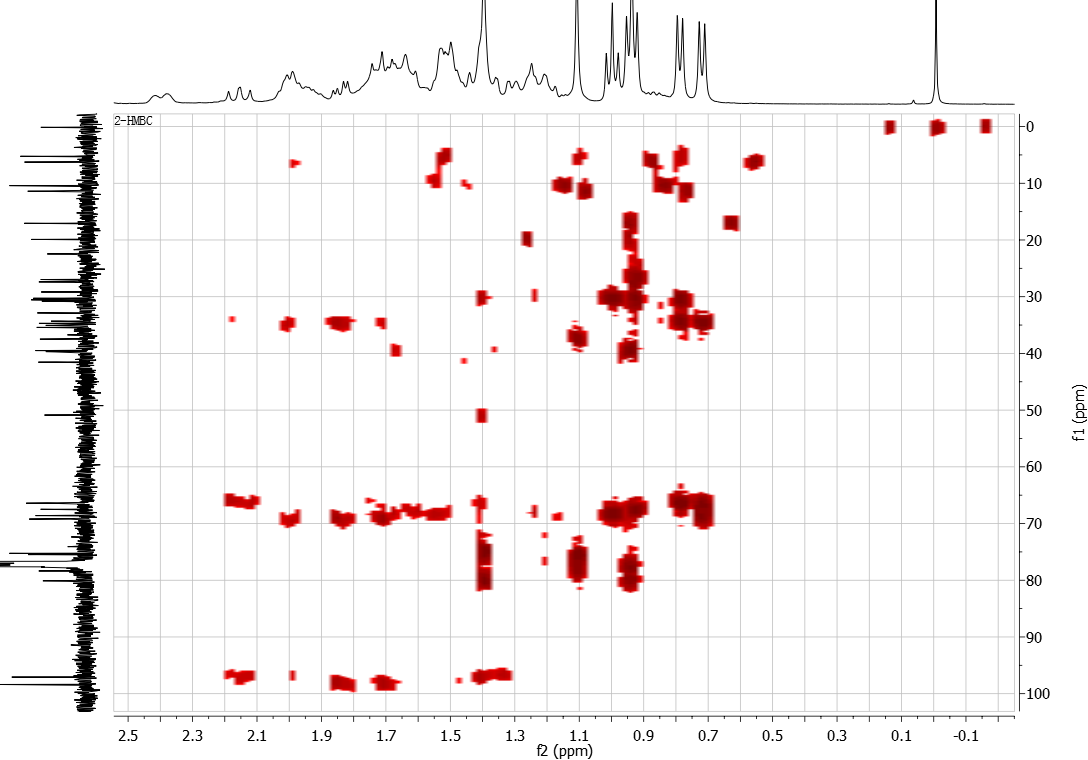


**Figure S17-3.** The HMBC spectrum of compound **2** in CDCl_3_ (400 MHz)


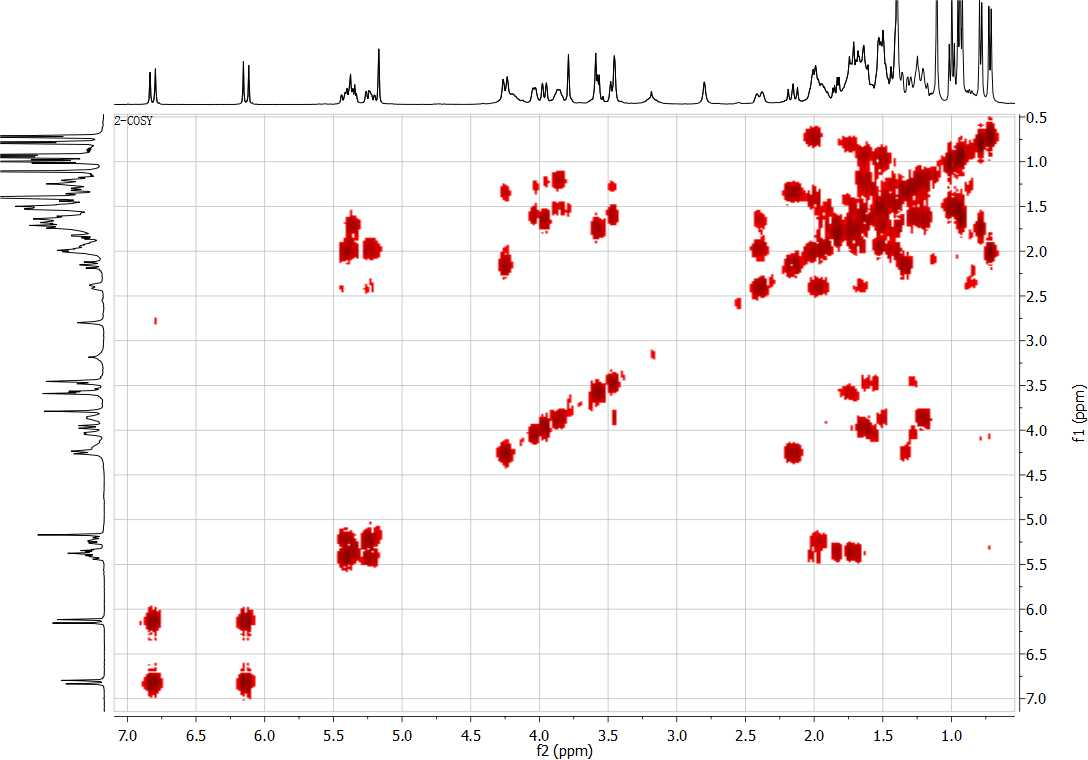


**Figure S18.** The 1H-1H COSY spectrum of compound **2** in CDCl_3_ (400 MHz)


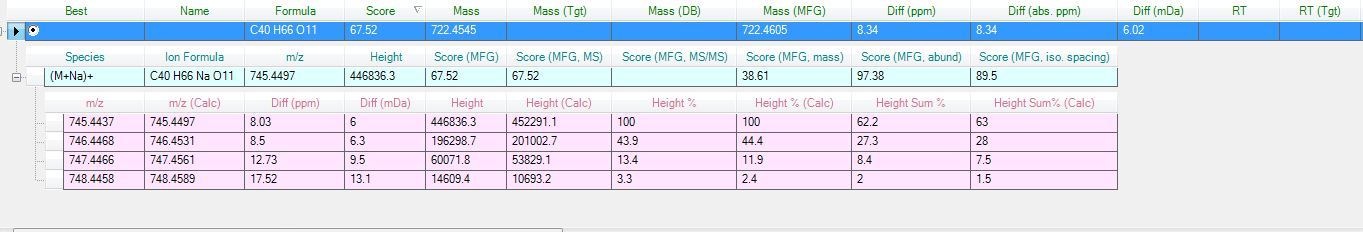

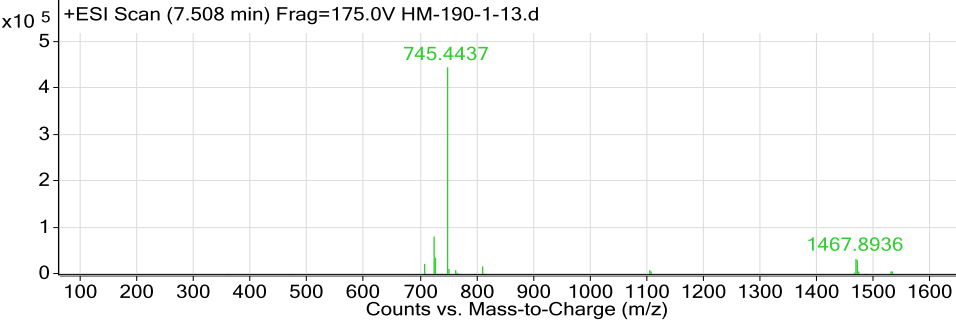


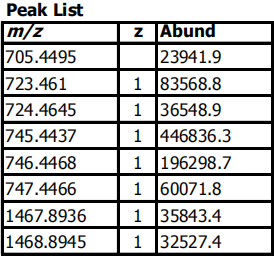


**Figure S19.** The HRESIMS data of compound **3**


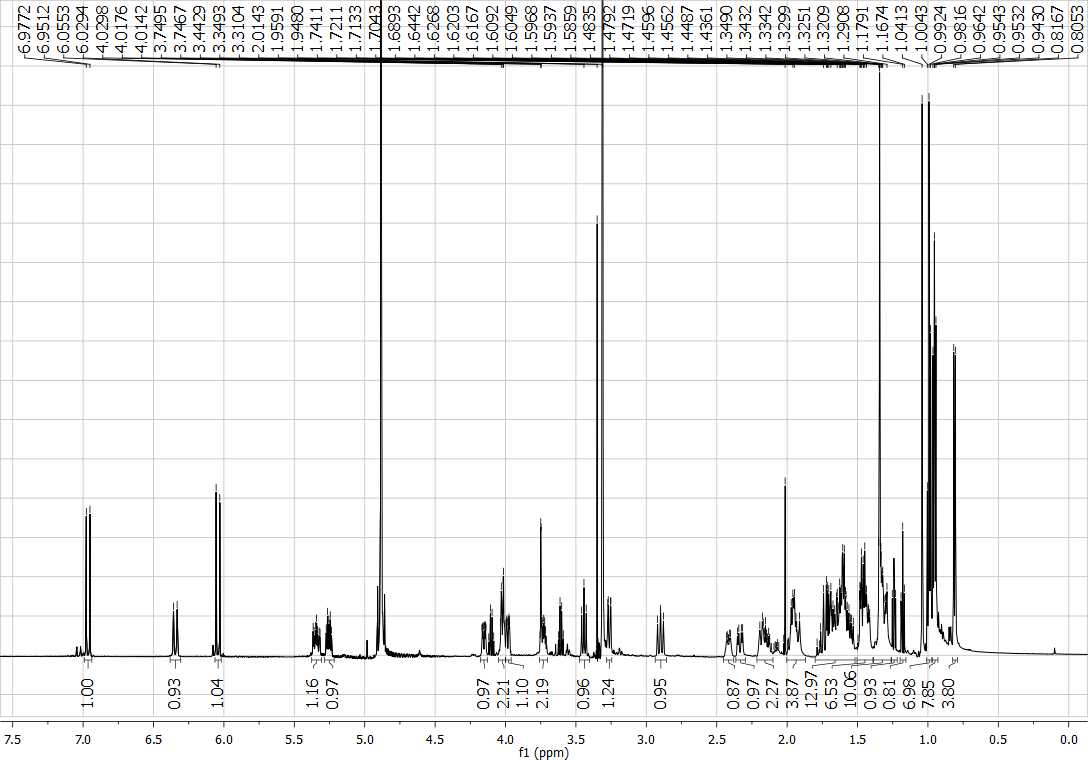


**Figure S20.** The 1H NMR spectrum of compound **3** in CD_3_OD (600 MHz)


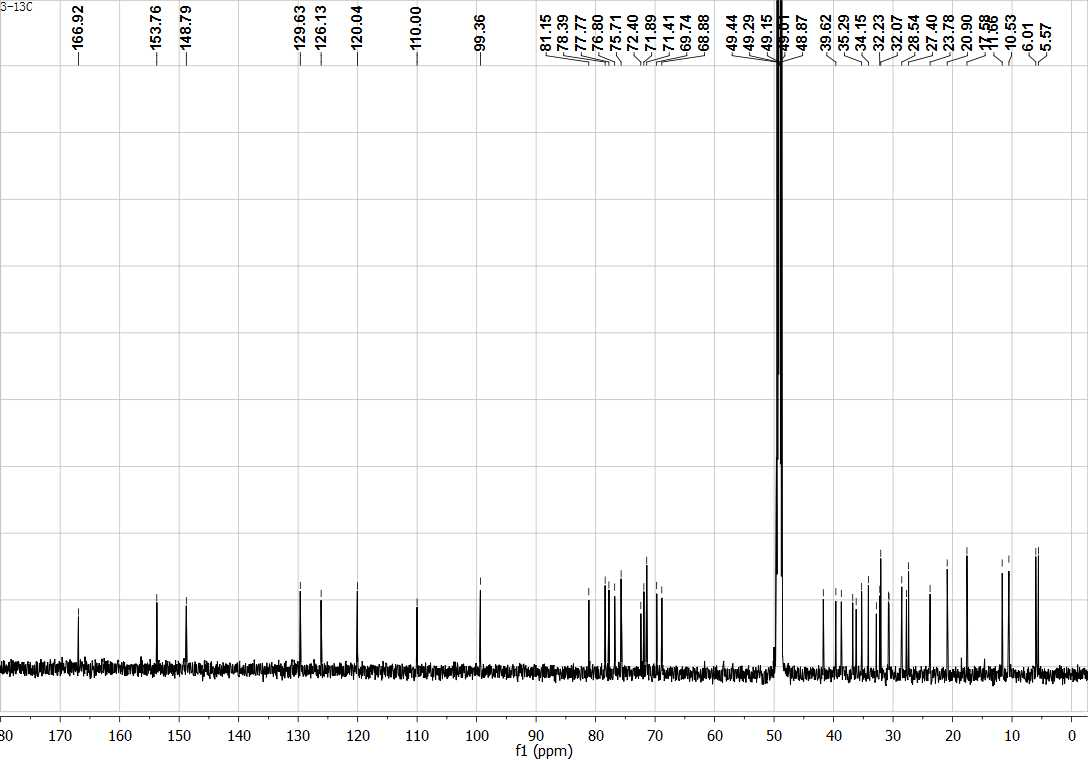


**Figure S21.** The 13C NMR spectrum of compound **3** in CD_3_OD (150 MHz)


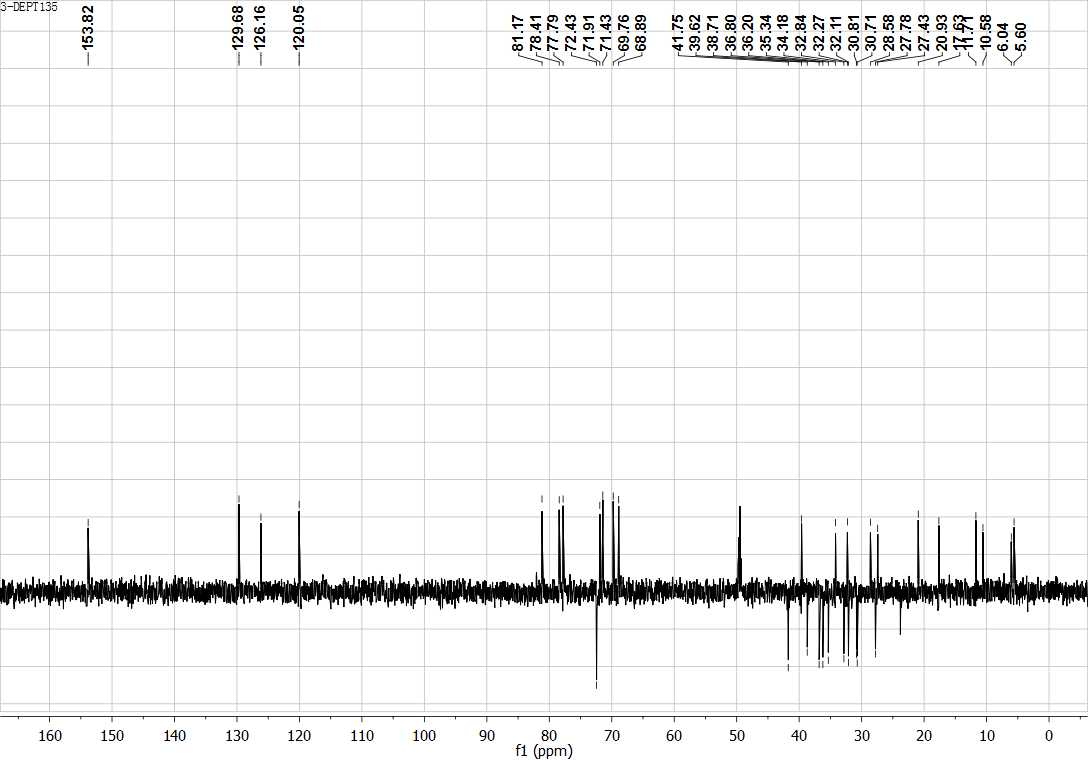


**Figure S22.** The DEPT135 spectrum of compound **3** in CD_3_OD (600 MHz)


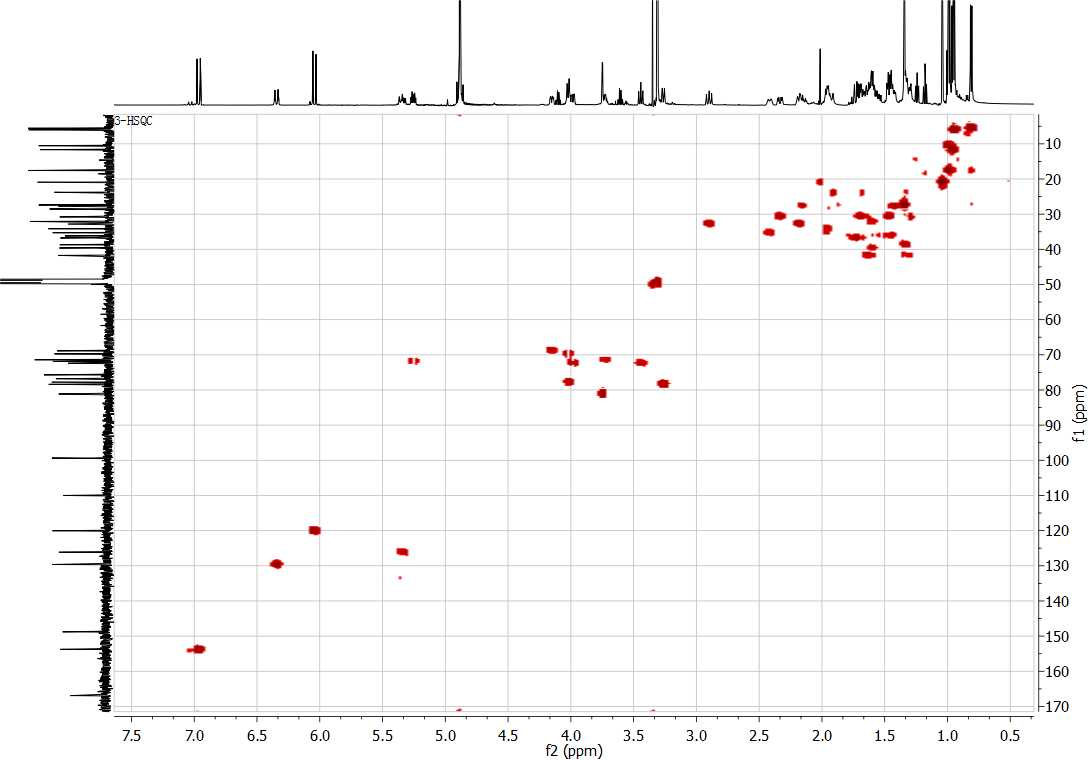


**Figure S23.** The HSQC spectrum of compound **3** in CD_3_OD (600 MHz)


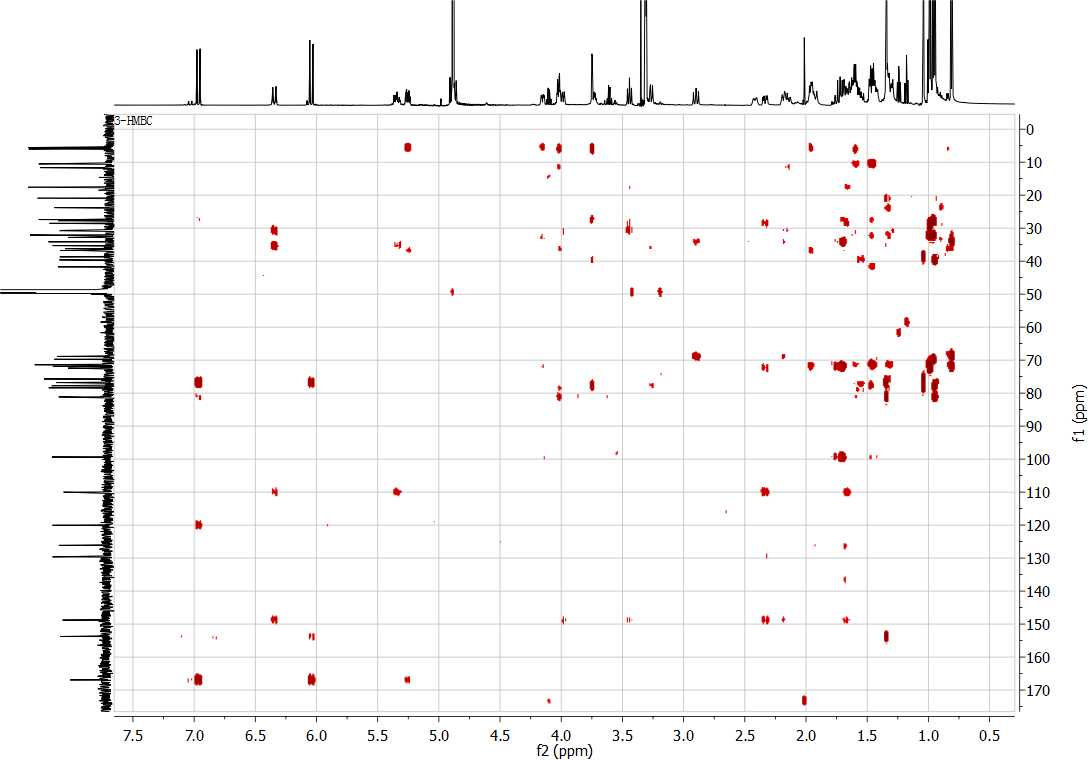


**Figure S24-1.** The HMBC spectrum of compound **3** in CD_3_OD (600 MHz)


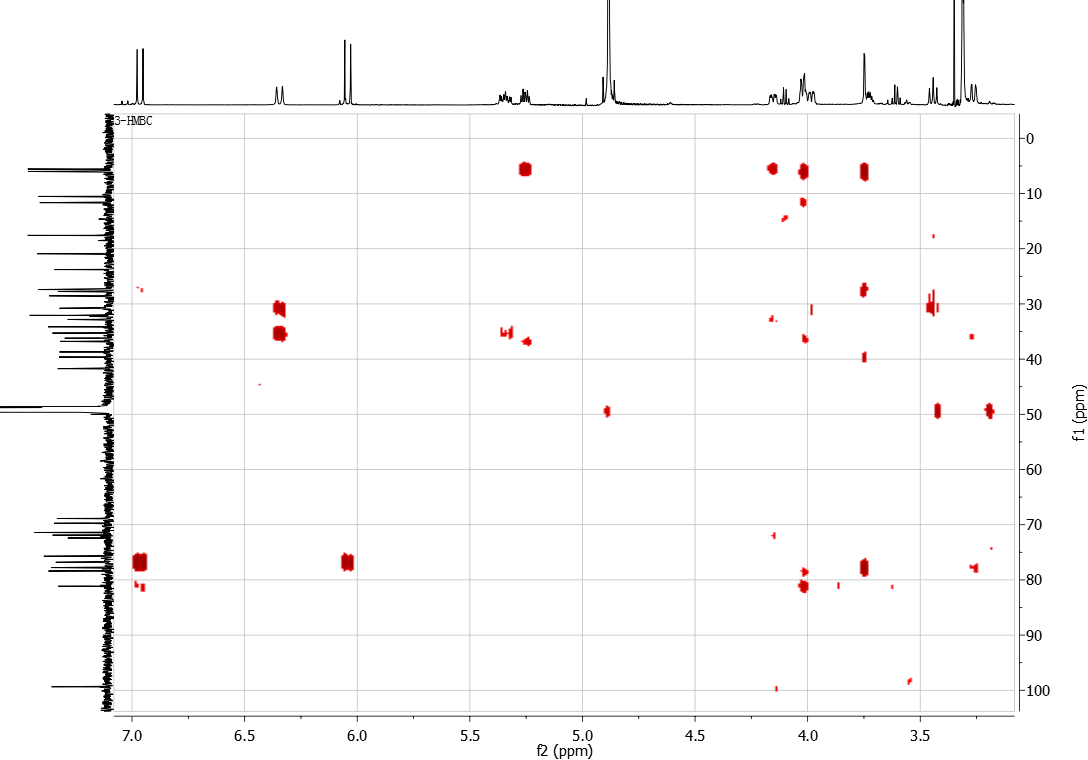


**Figure S24-2.** The HMBC spectrum of compound **3** in CD_3_OD (600 MHz)


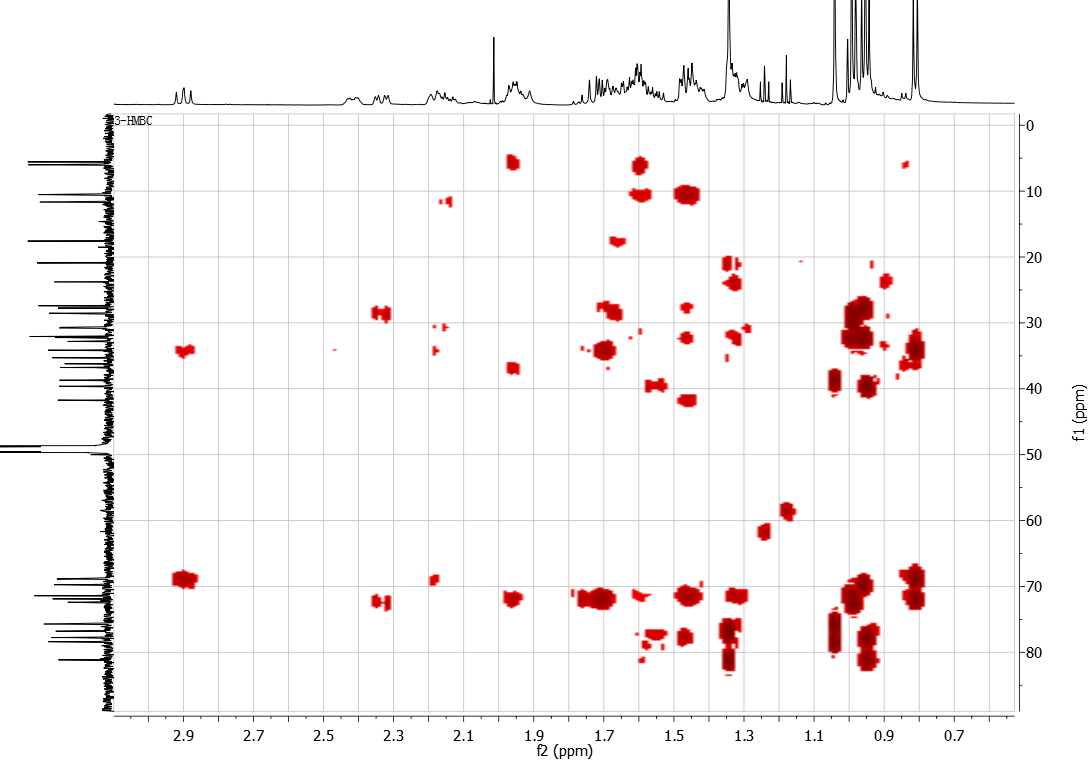


**Figure S24-3.** The HMBC spectrum of compound **3** in CD_3_OD (600 MHz)


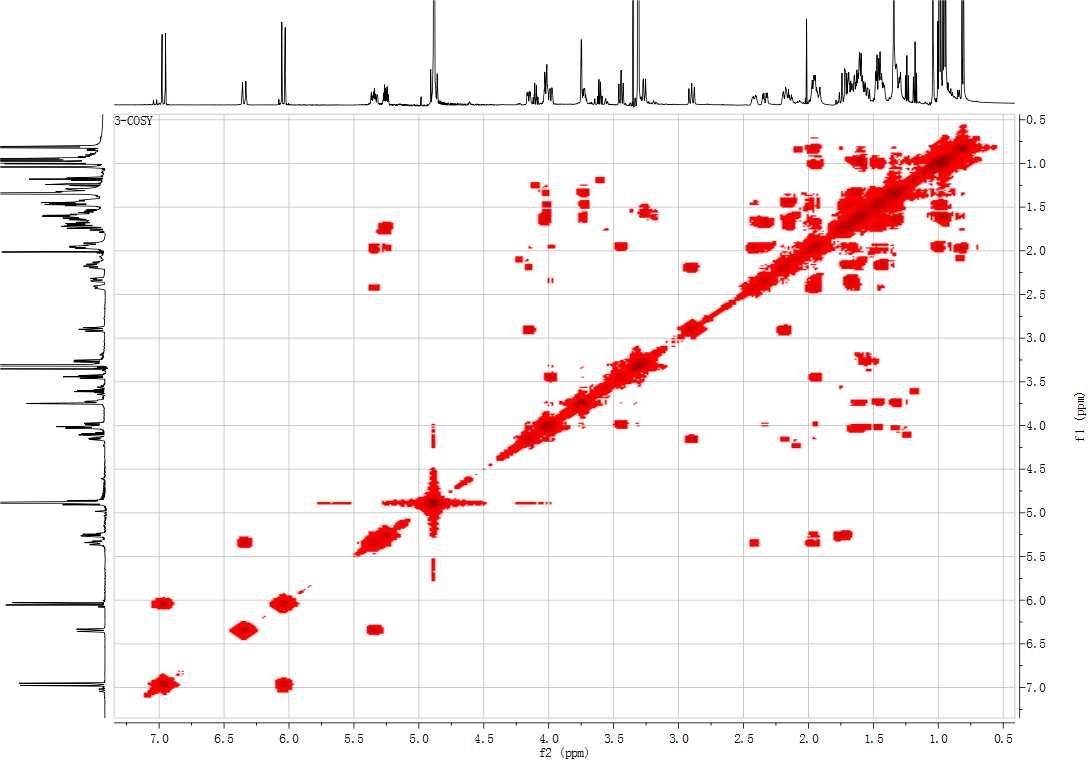
**Figure S25.** The 1H-1H COSY spectrum of compound **3** in CD_3_OD (600 MHz)

**Figure S26.** The maximum-parsimony tree shown the phylogenetic relationships between strain HM190 and related species of the genus *Streptomyces* based on 16S rRNA gene sequences.

**Figure S27.** The maximum-likelihood tree shown the phylogenetic relationships between strain HM190 and related species of the genus *Streptomyce*s based on 16S rRNA gene sequences.

**Table S1.** The clusters of orthologous groups (COG) classification of the complete genome of strain HM190.

| **First class** | **Second class** | **Class description** | **Gene number** |
| --- | --- | --- | --- |
| Information | A | RNA processing and modification | 1 |
| Information | B | Chromatin structure and dynamics | 3 |
| Metabolism | C | Energy production and conversion | 290 |
| Cellular | D | Cell cycle control, cell division and chromosome partitioning | 69 |
| Metabolism | E | Amino acid transport and metabolism | 478 |
| Metabolism | F | Nucleotide transport and metabolism | 123 |
| Metabolism | G | Carbohydrate transport and metabolism | 502 |
| Metabolism | H | Coenzyme transport and metabolism | 297 |
| Metabolism | I | Lipid transport and metabolism | 316 |
| Information | J | Translation, ribosomal structure and biogenesis | 261 |
| Information | K | Transcription | 613 |
| Information | L | Replication, recombination and repair | 151 |
| Cellular | M | Cell wall, membrane and envelope biogenesis | 258 |
| Cellular | N | Cell motility | 24 |
| Cellular | O | Posttranslational modification, protein turnover and chaperones | 208 |
| Metabolism | P | Inorganic ion transport and metabolism | 253 |
| Metabolism | Q | Secondary metabolites biosynthesis, transport and catabolism | 216 |
| Poorly | R | General function prediction only | 612 |
| Poorly | S | Function unknown | 257 |
| Cellular | T | Signal transduction mechanisms | 418 |
| Cellular | U | Intracellular trafficking, secretion and vesicular transport | 49 |
| Cellular | V | Defense mechanisms | 166 |
| Cellular | W | Extracellular structures | 10 |
| Metabolism | X | Mobilome: prophages and transposons | 80 |

**Table S2.** The antiSMASH-predicted BGCs for [strain](https://www.ezbiocloud.net/taxonomy?tn=Streptomyces heilongjiangensis)HM190.

| **BGC** | **Position** | | **Type** | **Predicted product** |
| --- | --- | --- | --- | --- |
|  | **From To** | |  |  |
| Cluster 1 | 130785 | 199361 | Non-ribosomal peptide synthetase cluster, other types of PKS cluster | 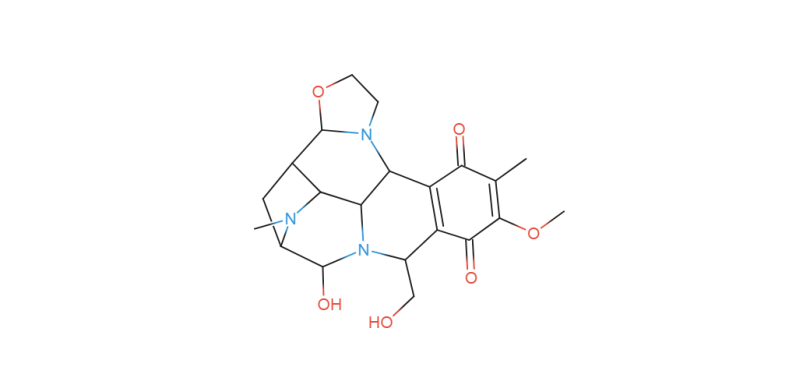 |
| Cluster 2 | 203880 | 243093 | Type III PKS (polyketide synthase) | Alkylresorcinol |
| Cluster 3 | 259375 | 324831 | Non-ribosomal peptide synthetase cluster | 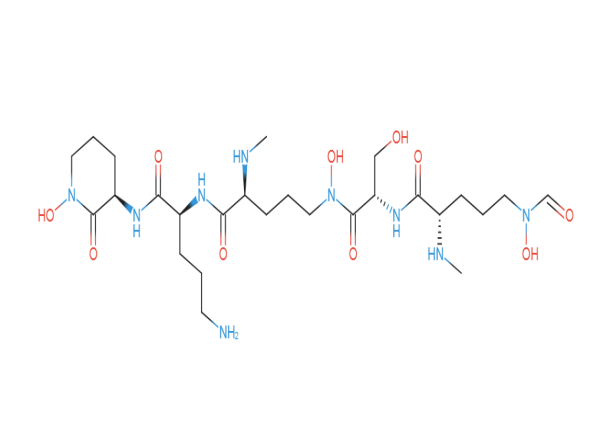 |
| Cluster 4 | 513690 | 578383 | Type I PKS, NRPS-like fragment | [Prejadomycin, rabelomycin, gaudimycin C, gaudimycin D, gaudimycin A](https://mibig.secondarymetabolites.org/go/BGC0000262/1) |
| Cluster 5 | 639615 | 765945 | Type I PKS | 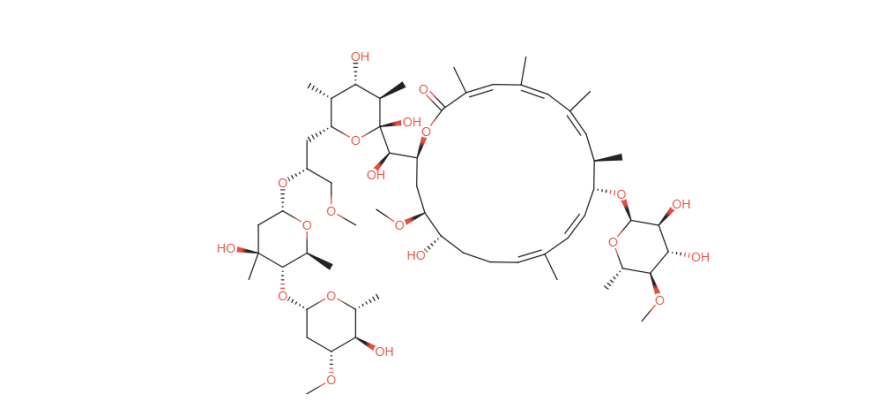 |
| Cluster 6 | 1638793 | 1680974 | NRPS-like fragment, Type II PKS | 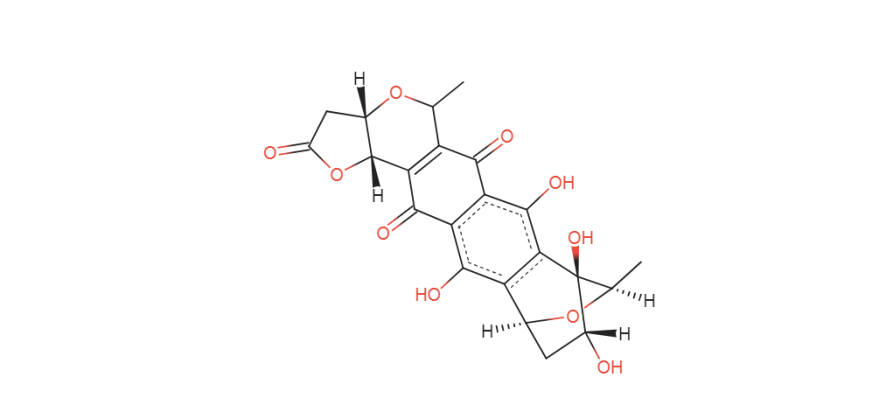 |
| Cluster 7 | 1895742 | 1906140 | Ectoine cluster | 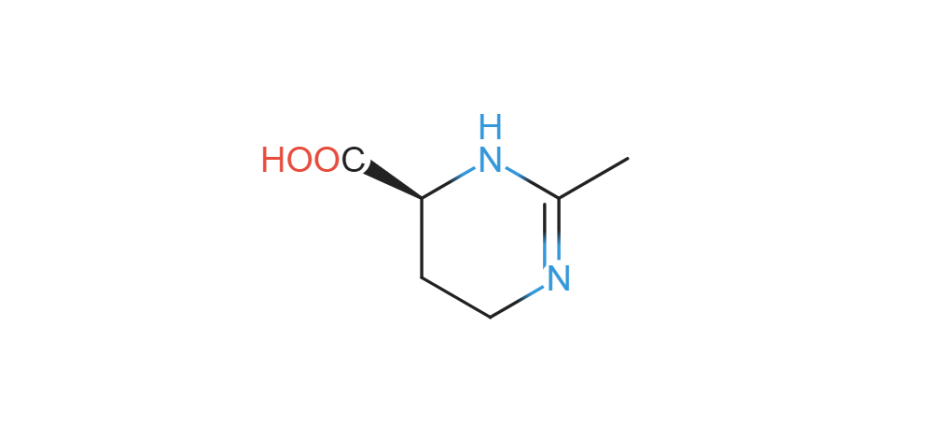 |
| Cluster 8 | 1926897 | 1946701 | Saccharide cluster (loose strictness, likely from primary metabolism) | 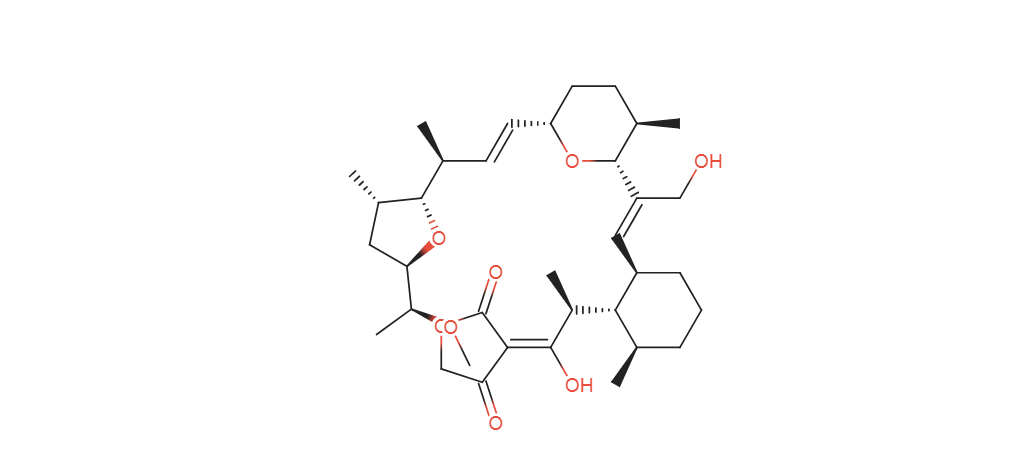 |
| Cluster 9 | 2325533 | 2353947 | Saccharide cluster (loose strictness, likely from primary metabolism) | [Acarviostatin I03, acarviostatin II03, acarviostatin III03, acarviostatin IV03](https://mibig.secondarymetabolites.org/go/BGC0000804/1) |
| Cluster 10 | 2668663 | 2751181 | Other types of PKS cluster, linear azol(in)e-containing peptides, [butyrolactone](https://docs.antismash.secondarymetabolites.org/glossary/#butyrolactone) cluster, type I PKS | 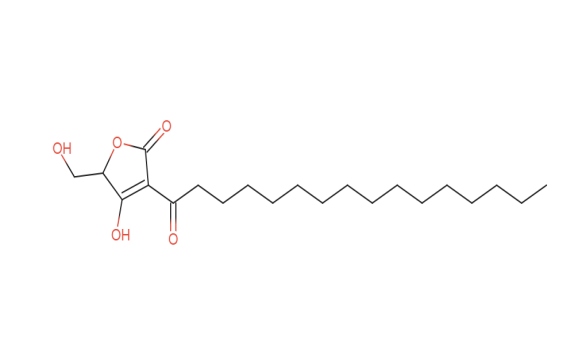 |
| Cluster 11 | 3042922 | 3053371 | [Melanin](https://docs.antismash.secondarymetabolites.org/glossary/#melanin) cluster,  saccharide cluster | 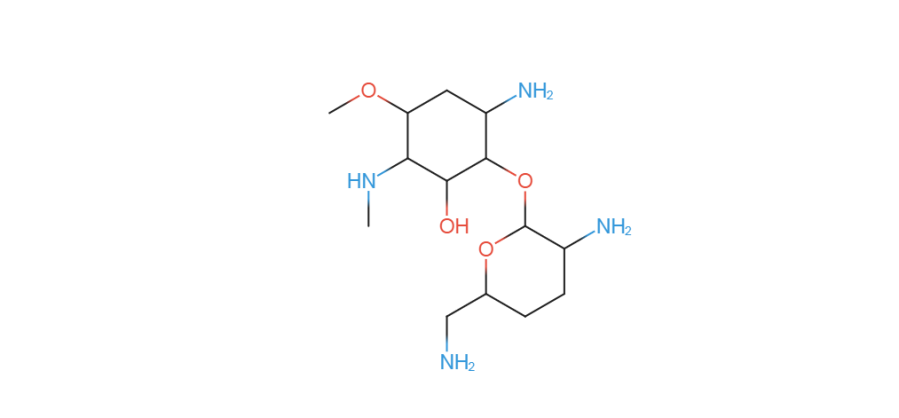 |
| Cluster 12 | 3186240 | 3196473 | [Siderophore](https://docs.antismash.secondarymetabolites.org/glossary/#siderophore) cluster | 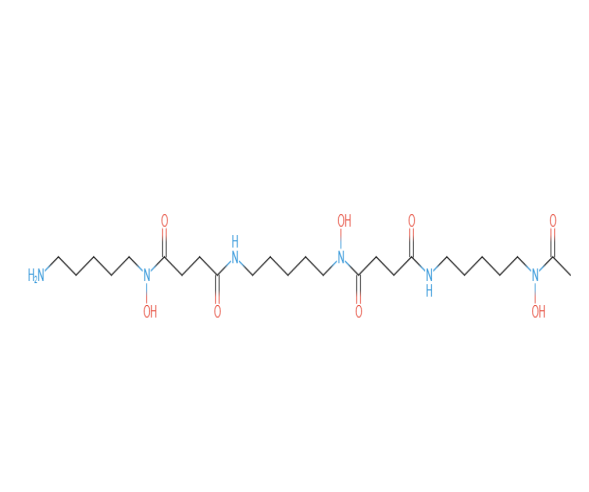 |
| Cluster 13 | 3289672 | 3300654 | Bacteriocin or other unspecified ribosomally synthesised and post-translationally modified peptide product (RiPP) cluster | 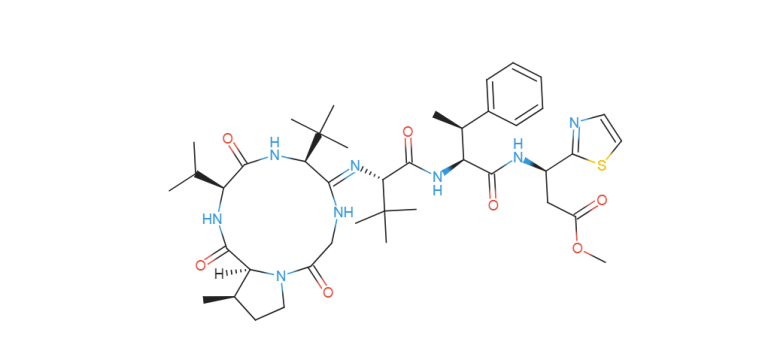 |
| Cluster 14 | 3542825 | 3615361 | Type II PKS | Hiroshidine |
| Cluster 15 | 4521420 | 4582890 | Non-ribosomal peptide synthetase cluster, type I PKS, [indole](https://docs.antismash.secondarymetabolites.org/glossary/#indole) cluster | 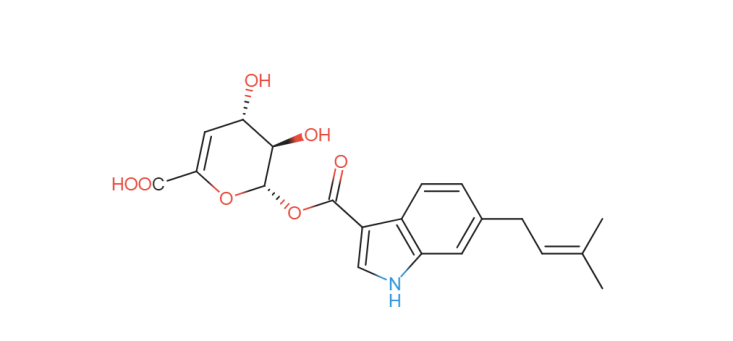 |
| Cluster 16 | 4610002 | 4651186 | Type III PKS | 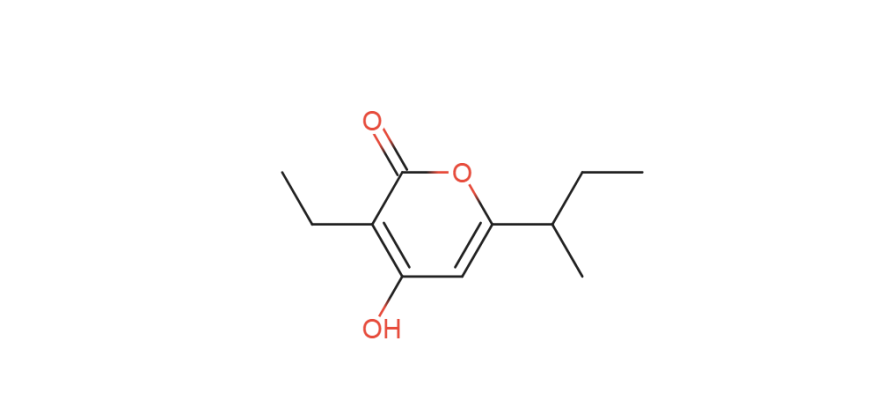 |
| Cluster 17 | 4855119 | 4884565 | Lanthipeptide cluster, lasso peptide cluster | Citrulassin E |
| Cluster 18 | 4897394 | 4918563 | Indole cluster | 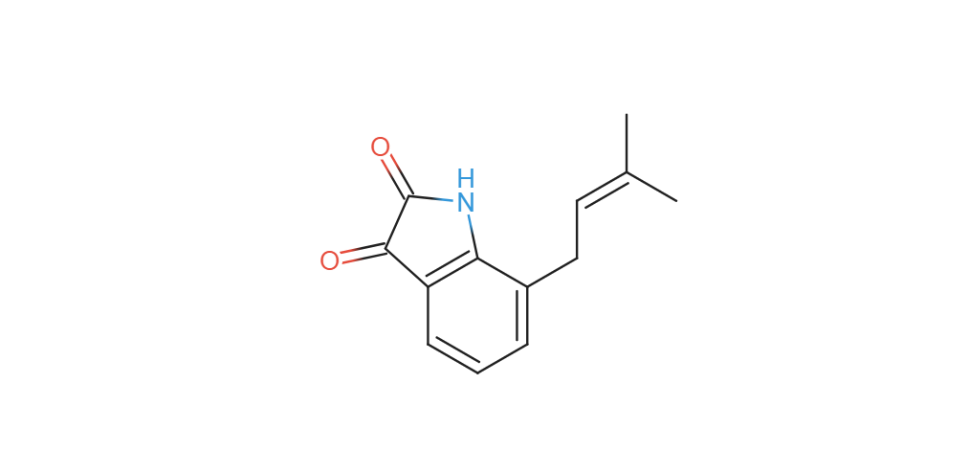 |
| Cluster 19 | 5116608 | 5137912 | Cluster containing a halogenase and thus potentially generating a halogenated product | Halogenide |
| Cluster 20 | 5515693 | 5555679 | Saccharide cluster (loose strictness, likely from primary metabolism) | 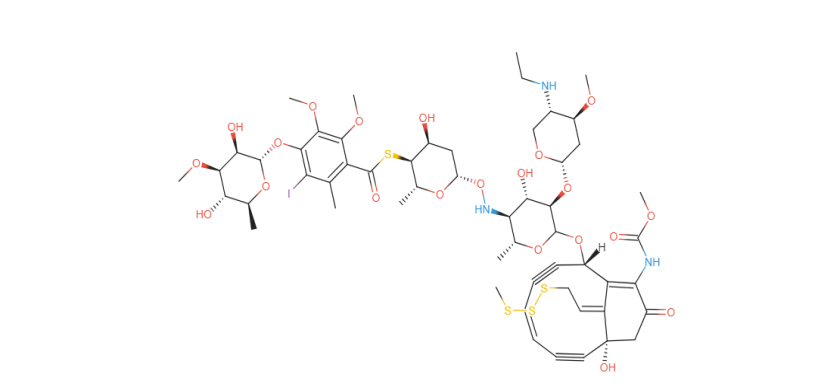 |
| Cluster 21 | 5618752 | 5684789 | Non-ribosomal peptide synthetase cluster | 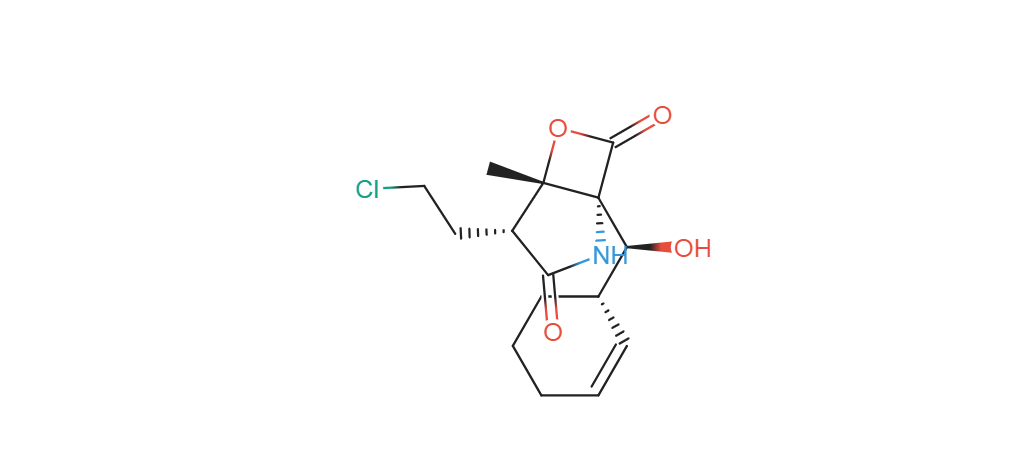 |
| Cluster 22 | 6053546 | 6127384 | Saccharide cluster (loose strictness, likely from primary metabolism) | 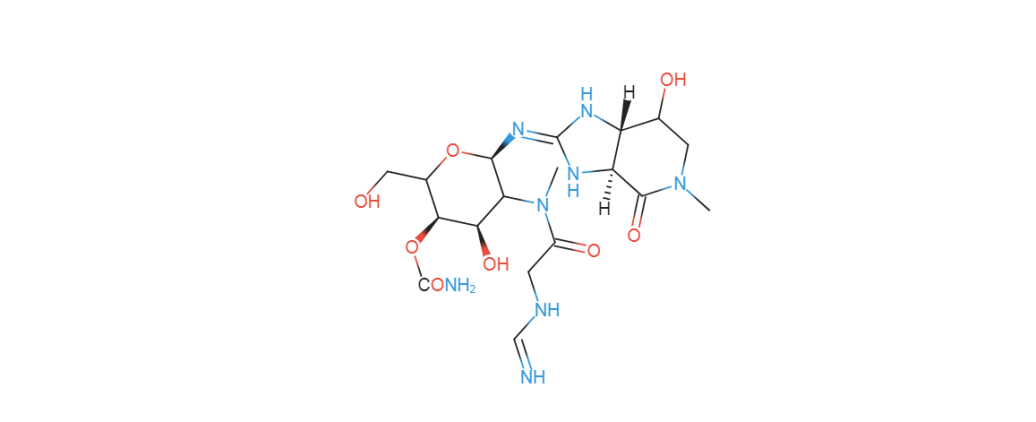 |
| Cluster 23 | 6443008 | 6496695 | *β*-lactone containing protease inhibitor, NRPS-like fragment, type I PKS, [siderophore](https://docs.antismash.secondarymetabolites.org/glossary/#siderophore) cluster | Carrimycin |
| Cluster 24 | 6641850 | 6662850 | [Terpene](https://docs.antismash.secondarymetabolites.org/glossary/#terpene) | 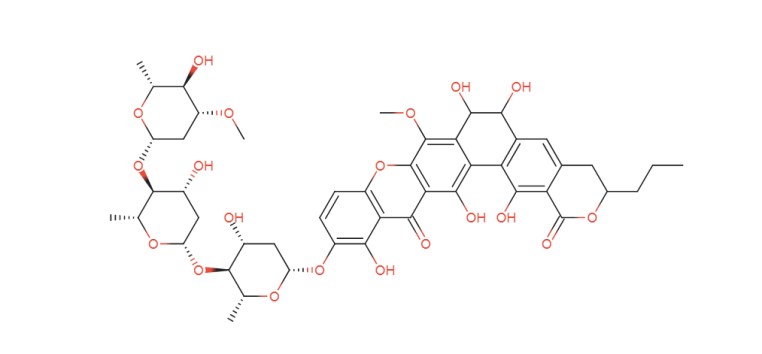 |
| Cluster 25 | 6742693 | 6754117 | Bacteriocin or other unspecified ribosomally synthesised and post-translationally modified peptide product (RiPP) cluster | [Bacteriocin](https://docs.antismash.secondarymetabolites.org/glossary/#bacteriocin) |
| Cluster 26 | 6810136 | 6830882 | [Terpene](https://docs.antismash.secondarymetabolites.org/glossary/#terpene) | 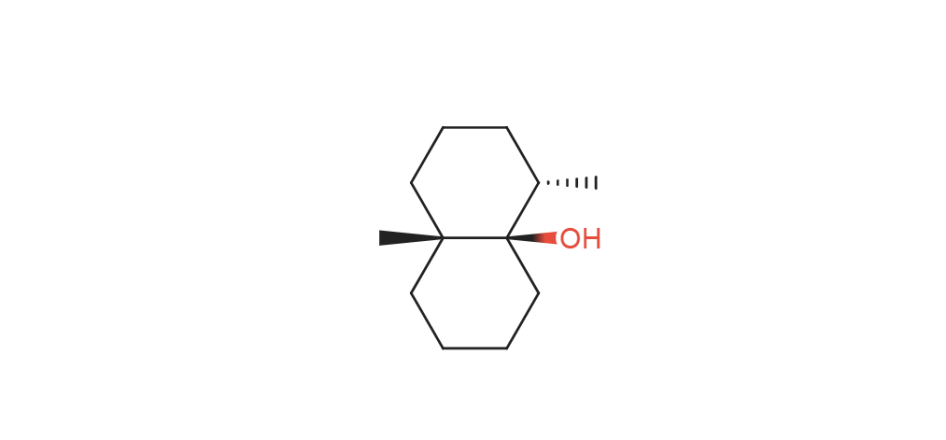 |
| Cluster 27 | 7050005 | 7063252 | Siderophore cluster | [Siderophore](https://docs.antismash.secondarymetabolites.org/glossary/#siderophore) |
| Cluster 28 | 7064839 | 7087363 | Linear azol(in)e-containing peptides | [LAP](https://docs.antismash.secondarymetabolites.org/glossary/#lap) |
| Cluster 29 | 7400389 | 7421792 | Saccharide cluster (loose strictness, likely from primary metabolism) | 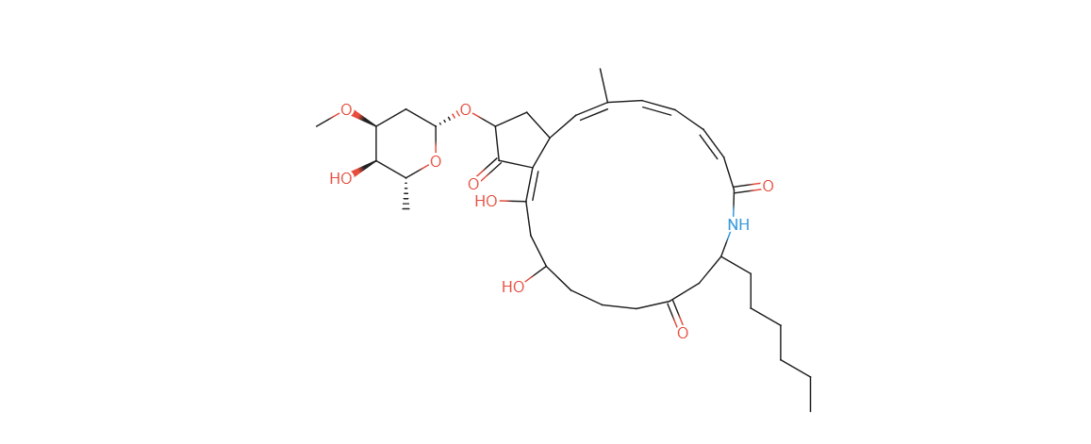 |
| Cluster 30 | 7569553 | 7596224 | [Terpene](https://docs.antismash.secondarymetabolites.org/glossary/#terpene) | 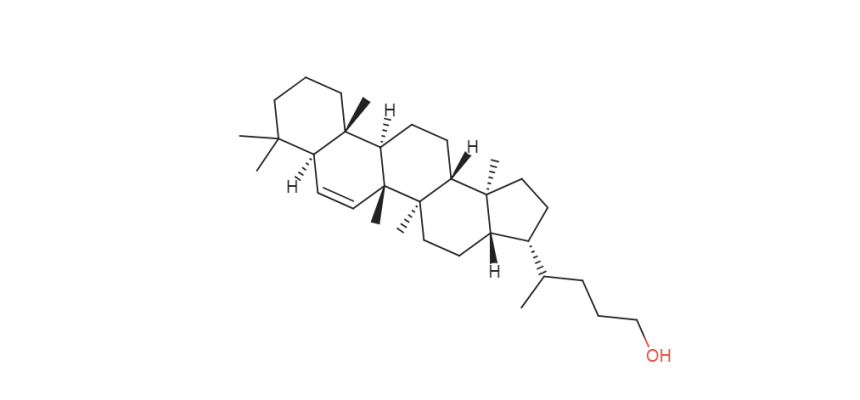 |

**Table S3.** Fractional atomic coordinates (×10^4^) and equivalent isotropic displacement parameters (Å^2^×10^3^) for compound **1** by the X-ray crystallography.

| **Atom** | ***x*** | ***y*** | ***z*** | **U (eq)** |
| --- | --- | --- | --- | --- |
| O1 | 7618(4) | 7025.3(18) | 6971.8(19) | 45.5(10) |
| O2 | 8976(5) | 7544(3) | 7690(2) | 88.7(19) |
| O3 | 4820(4) | 7113(2) | 8714.8(19) | 52.4(11) |
| O4 | 2163(4) | 7240.8(19) | 8232.5(19) | 47.3(10) |
| O5 | 1193(4) | 8436(2) | 7804.4(19) | 52.3(11) |
| O6 | 1006(4) | 9230.3(19) | 6860.0(19) | 46.4(10) |
| O7 | 2540(4) | 8581.2(16) | 6062.4(16) | 41.2(10) |
| O8 | 2147(4) | 9897.8(17) | 5984(2) | 50.1(11) |
| O9 | 8699(5) | 8540.5(17) | 4422.6(18) | 48.8(11) |
| O10 | 8321(4) | 7625.5(16) | 5249.3(18) | 41.0(9) |
| O11 | 9888(4) | 6797.1(17) | 5455.8(18) | 42.8(10) |
| O12 | 13218(5) | 7640.6(19) | 5351(2) | 58.5(12) |
| O13 | 10690(4) | 8795.4(17) | 5031.9(18) | 45.0(10) |
| C1 | 7918(7) | 7263(3) | 7532(3) | 57.4(18) |
| C2 | 6854(7) | 7192(3) | 7938(3) | 53.2(17) |
| C3 | 5587(7) | 7008(3) | 7722(3) | 47.9(16) |
| C4 | 4442(6) | 6924(3) | 8105(3) | 44.9(15) |
| C5 | 3194(6) | 7316(3) | 7841(3) | 38.5(14) |
| C6 | 3443(6) | 8010(2) | 7665(3) | 37.1(13) |
| C7 | 2124(6) | 8320(3) | 7362(2) | 37.5(14) |
| C8 | 2313(6) | 8975(3) | 7075(3) | 38.0(14) |
| C9 | 3166(6) | 8980(2) | 6541(3) | 36.6(13) |
| C10 | 3414(6) | 9647(2) | 6288(3) | 39.4(14) |
| C11 | 4373(7) | 9608(3) | 5785(3) | 48.3(16) |
| C12 | 4514(7) | 10238(3) | 5444(4) | 69(2) |
| C13 | 5241(7) | 10162(4) | 4875(4) | 73(2) |
| C14 | 6750(7) | 10055(3) | 5014(3) | 51.5(17) |
| C15 | 7486(7) | 9709(3) | 4671(3) | 49.5(16) |
| C16 | 8966(6) | 9619(2) | 4781(3) | 40.3(14) |
| C17 | 9288(6) | 8901(2) | 4935(3) | 37.1(14) |
| C18 | 8700(6) | 8714(2) | 5525(3) | 39.9(14) |
| C19 | 8920(6) | 8034(2) | 5742(3) | 35.6(13) |
| C20 | 8260(6) | 7918(3) | 6330(3) | 40.9(14) |
| C21 | 8445(6) | 7213(3) | 6500(3) | 43.6(15) |
| C22 | 7904(6) | 6778(3) | 5945(3) | 48.0(16) |
| C23 | 8493(7) | 6954(2) | 5372(3) | 45.4(15) |
| C24 | 7760(7) | 6626(3) | 4818(3) | 58.3(18) |
| C25 | 8444(8) | 6696(3) | 4252(3) | 66(2) |
| C26 | 9936(7) | 6507(3) | 4380(3) | 55.3(18) |
| C27 | 10620(6) | 6885(3) | 4925(3) | 45.3(16) |
| C28 | 12027(6) | 6654(3) | 5130(3) | 43.4(15) |
| C29 | 12775(6) | 7070(3) | 5619(3) | 47.7(16) |
| C30 | 14023(7) | 6742(3) | 5956(3) | 57.4(18) |
| C31 | 13676(8) | 6174(3) | 6329(3) | 64(2) |
| C32 | 11392(7) | 8951(3) | 4506(3) | 49.7(17) |
| C33 | 11203(7) | 9644(3) | 4337(3) | 51.7(17) |
| C34 | 9702(7) | 9809(3) | 4227(3) | 46.1(16) |
| C35 | 4104(7) | 6202(3) | 8090(3) | 57.1(18) |
| C36 | 4101(7) | 8418(3) | 8201(3) | 56.0(18) |
| C37 | 3929(8) | 10103(3) | 6805(3) | 64(2) |
| C38 | 6793(6) | 8129(3) | 6292(3) | 53.9(17) |
| C39 | 11893(8) | 9798(3) | 3764(3) | 62.2(19) |

**Table S4.** Bond lengths (Å) for compound **1** by the X-ray crystallography.

| **Atom** | **Atom** | **Length** |
| --- | --- | --- |
| O1 | C1 | 1.330(8) |
| O1 | C21 | 1.447(7) |
| O2 | C1 | 1.225(8) |
| O3 | C4 | 1.408(7) |
| O4 | C5 | 1.420(6) |
| O5 | C7 | 1.436(6) |
| O6 | C8 | 1.439(7) |
| O7 | C9 | 1.431(6) |
| O8 | C10 | 1.461(7) |
| O9 | C17 | 1.428(6) |
| O10 | C19 | 1.456(6) |
| O10 | C23 | 1.442(6) |
| O11 | C23 | 1.421(7) |
| O11 | C27 | 1.453(7) |
| O12 | C29 | 1.427(7) |
| O13 | C17 | 1.409(7) |
| O13 | C32 | 1.452(7) |
| C1 | C2 | 1.467(9) |
| C2 | C3 | 1.356(9) |
| C3 | C4 | 1.500(8) |
| C4 | C5 | 1.551(8) |
| C4 | C35 | 1.553(8) |
| C5 | C6 | 1.535(8) |
| C6 | C7 | 1.550(8) |
| C6 | C36 | 1.542(8) |
| C7 | C8 | 1.535(7) |
| C8 | C9 | 1.521(8) |
| C9 | C10 | 1.538(8) |
| C10 | C11 | 1.541(8) |
| C10 | C37 | 1.531(8) |
| C11 | C12 | 1.535(8) |
| C12 | C13 | 1.519(10) |
| C13 | C14 | 1.518(9) |
| C14 | C15 | 1.324(9) |
| C15 | C16 | 1.481(9) |
| C16 | C17 | 1.570(7) |
| C16 | C34 | 1.540(8) |
| C17 | C18 | 1.530(8) |
| C18 | C19 | 1.513(7) |
| C19 | C20 | 1.532(8) |
| C20 | C21 | 1.533(8) |
| C20 | C38 | 1.522(8) |
| C21 | C22 | 1.570(9) |
| C22 | C23 | 1.492(8) |
| C23 | C24 | 1.515(9) |
| C24 | C25 | 1.487(10) |
| C25 | C26 | 1.535(10) |
| C26 | C27 | 1.533(9) |
| C27 | C28 | 1.505(9) |
| C28 | C29 | 1.515(8) |
| C29 | C30 | 1.538(9) |
| C30 | C31 | 1.510(10) |
| C32 | C33 | 1.509(8) |
| C33 | C34 | 1.529(9) |
| C33 | C39 | 1.533(9) |

**Table S5.** Bond angles (˚) for compound **1** by the X-ray crystallography.

| **Atom** | **Atom** | **Atom** | **Angle** |
| --- | --- | --- | --- |
| C1 | O1 | C21 | 118.1(5) |
| C23 | O10 | C19 | 114.1(4) |
| C23 | O11 | C27 | 115.9(5) |
| C17 | O13 | C32 | 113.6(5) |
| O1 | C1 | C2 | 114.8(6) |
| O2 | C1 | O1 | 122.8(6) |
| O2 | C1 | C2 | 122.3(7) |
| C3 | C2 | C1 | 121.6(6) |
| C2 | C3 | C4 | 125.0(6) |
| O3 | C4 | C3 | 111.1(5) |
| O3 | C4 | C5 | 108.9(5) |
| O3 | C4 | C35 | 109.3(5) |
| C3 | C4 | C5 | 110.9(5) |
| C3 | C4 | C35 | 106.3(5) |
| C5 | C4 | C35 | 110.4(5) |
| O4 | C5 | C4 | 108.6(4) |
| O4 | C5 | C6 | 113.9(4) |
| C6 | C5 | C4 | 117.0(5) |
| C5 | C6 | C7 | 110.7(5) |
| C5 | C6 | C36 | 113.8(5) |
| C36 | C6 | C7 | 111.4(4) |
| O5 | C7 | C6 | 111.2(4) |
| O5 | C7 | C8 | 104.1(4) |
| C8 | C7 | C6 | 114.6(5) |
| O6 | C8 | C7 | 108.7(5) |
| O6 | C8 | C9 | 107.9(5) |
| C9 | C8 | C7 | 115.2(4) |
| O7 | C9 | C8 | 109.1(4) |
| O7 | C9 | C10 | 110.1(5) |
| C8 | C9 | C10 | 114.1(4) |
| O8 | C10 | C9 | 109.2(5) |
| O8 | C10 | C11 | 105.3(5) |
| O8 | C10 | C37 | 108.4(5) |
| C9 | C10 | C11 | 110.3(4) |
| C37 | C10 | C9 | 111.0(5) |
| C37 | C10 | C11 | 112.4(5) |
| C12 | C11 | C10 | 113.7(5) |
| C13 | C12 | C11 | 112.8(5) |
| C14 | C13 | C12 | 113.7(7) |
| C15 | C14 | C13 | 124.4(7) |
| C14 | C15 | C16 | 125.6(7) |
| C15 | C16 | C17 | 109.5(5) |
| C15 | C16 | C34 | 113.1(5) |
| C34 | C16 | C17 | 108.4(5) |
| O9 | C17 | C16 | 106.5(5) |
| O9 | C17 | C18 | 111.5(5) |
| O13 | C17 | O9 | 110.5(4) |
| O13 | C17 | C16 | 111.1(4) |
| O13 | C17 | C18 | 107.3(5) |
| C18 | C17 | C16 | 110.0(4) |
| C19 | C18 | C17 | 117.0(4) |
| O10 | C19 | C18 | 106.8(4) |
| O10 | C19 | C20 | 110.6(4) |
| C18 | C19 | C20 | 110.8(4) |
| C19 | C20 | C21 | 108.0(4) |
| C38 | C20 | C19 | 114.0(5) |
| C38 | C20 | C21 | 112.6(5) |
| O1 | C21 | C20 | 112.0(4) |
| O1 | C21 | C22 | 102.9(5) |
| C20 | C21 | C22 | 110.5(5) |
| C23 | C22 | C21 | 112.2(5) |
| O10 | C23 | C22 | 110.5(5) |
| O10 | C23 | C24 | 105.0(5) |
| O11 | C23 | O10 | 110.2(4) |
| O11 | C23 | C22 | 107.6(5) |
| O11 | C23 | C24 | 112.1(5) |
| C22 | C23 | C24 | 111.4(5) |
| C25 | C24 | C23 | 113.5(6) |
| C24 | C25 | C26 | 110.2(6) |
| C27 | C26 | C25 | 110.3(5) |
| O11 | C27 | C26 | 109.9(5) |
| O11 | C27 | C28 | 104.6(5) |
| C28 | C27 | C26 | 112.7(5) |
| C27 | C28 | C29 | 113.3(5) |
| O12 | C29 | C28 | 110.1(5) |
| O12 | C29 | C30 | 107.7(5) |
| C28 | C29 | C30 | 113.1(5) |
| C31 | C30 | C29 | 113.2(6) |
| O13 | C32 | C33 | 110.8(5) |
| C32 | C33 | C34 | 110.6(5) |
| C32 | C33 | C39 | 110.5(5) |
| C34 | C33 | C39 | 110.0(6) |
| C33 | C34 | C16 | 111.0(5) |
